# Reactivity of a Dinuclear Pd^I^ Complex [Pd_2_(μ-PPh_2_)(μ_2_-OAc)(PPh_3_)_2_] with PPh_3_: Implications for Cross-Coupling Catalysis Using the Ubiquitous Pd(OAc)_2_/nPPh_3_ Catalyst System

**DOI:** 10.1021/acs.organomet.1c00347

**Published:** 2021-08-19

**Authors:** Neil W. J. Scott, Mark J. Ford, David R. Husbands, Adrian C. Whitwood, Ian J. S. Fairlamb

**Affiliations:** † Department of Chemistry, University of York, Heslington, York, North Yorkshire YO10 5DD, United Kingdom; ‡ Bayer AG, Alfred-Nobel-Strasse 50, 40789 Monheim, Germany

## Abstract

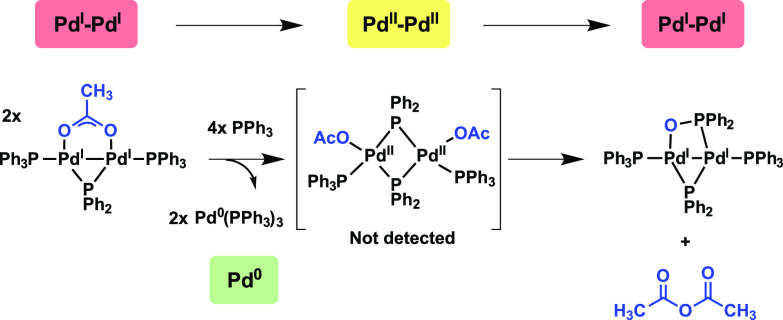

[Pd^I^_2_(μ-PPh_2_)(μ_2_-OAc)(PPh_3_)_2_] is the reduction product of Pd^II^(OAc)_2_(PPh_3_)_2_, generated by reaction of ‘Pd(OAc)_2_’ with two equivalents of PPh_3_. Here, we report that the reaction of [Pd^I^_2_(μ-PPh_2_)(μ_2_-OAc)(PPh_3_)_2_] with PPh_3_ results in a nuanced disproportionation reaction, forming [Pd^0^(PPh_3_)_3_] and a phosphinito-bridged Pd^I^-dinuclear complex, namely [Pd^I^_2_(μ-PPh_2_){κ_2_-P,O-μ-P(O)Ph_2_}(κ-PPh_3_)_2_]. The latter complex is proposed to form by abstraction of an oxygen atom from an acetate ligand at Pd. A mechanism for the formal reduction of a putative Pd^II^ disproportionation species to the observed Pd^I^ complex is postulated. Upon reaction of the mixture of [Pd^0^(PPh)_3_] and [Pd^I^_2_(μ-PPh_2_){κ_2_-P,O-μ-P(O)Ph_2_}(κ-PPh_3_)_2_] with 2-bromopyridine, the former Pd^0^ complex undergoes a fast oxidative addition reaction, while the latter dinuclear Pd^I^ complex converts slowly to a tripalladium cluster, of the type [Pd_3_(μ-X)(μ-PPh_2_)_2_(PPh_3_)_3_]X, with an overall 4/3 oxidation state *per* Pd. Our findings reveal complexity associated with the precatalyst activation step for the ubiquitous ‘Pd(OAc)_2_’/nPPh_3_ catalyst system, with implications for cross-coupling catalysis.

Pd^I^ dinuclear complexes are increasingly being adopted as effective and distinctive cross-coupling precatalysts.^1−8^ The Schoenebeck group has demonstrated that such complexes, and their derivatives, display unique reactivity, particularly with respect to controllable chemoselectivity in cross-coupling reactions, which has very recently been reviewed.^9^ Building on the important work on the activation of ‘Pd(OAc)_2_’ by Amatore and Jutand,^10−12^ our group recently discovered that the phosphido-bridged Pd^I^-dinuclear complex [Pd^I^_2_(μ-PPh_2_)(μ_2_-OAc)(PPh_3_)_2_] **1** forms via *trans*-Pd^II^(OAc)_2_(PPh_3_)_2_ in the reaction between [Pd^II^_3_(OAc)_6_] and exactly 6 equiv of PPh_3_ (where the Pd:PPh_3_ = 1:2). This is a precatalytic Pd:PPh_3_ ratio often employed as an effective catalyst system in synthetic chemistry applications. Recently, dinuclear Pd^I^ complexes have been shown to form by reaction between Pd(OAc)_2_ and dialkylbiaryl phosphines.^13−15^ As part of an investigation into its role in cross-coupling catalysis, the reactivity of **1** with electrophilic organohalides was conducted.^16^ [Pd^I^_2_(μ-PPh_2_)(μ_2_-OAc)(PPh_3_)_2_] **1** was found to activate organohalides at room temperature to afford tripalladium clusters, of the type [Pd_3_(μ-X)(μ-PPh_2_)_2_(PPh_3_)_3_]X, in addition to more commonly anticipated oxidative addition Pd^II^ products (Figure 1a). The finding, while intriguing for catalytic cross-coupling, is underpinned by a significant history of [Pd_3_(X)(PPh_2_)_2_(PPh_3_)_3_]X clusters dating back to their synthesis and characterization in the late 1960s.^17−20^

**Figure 1:**
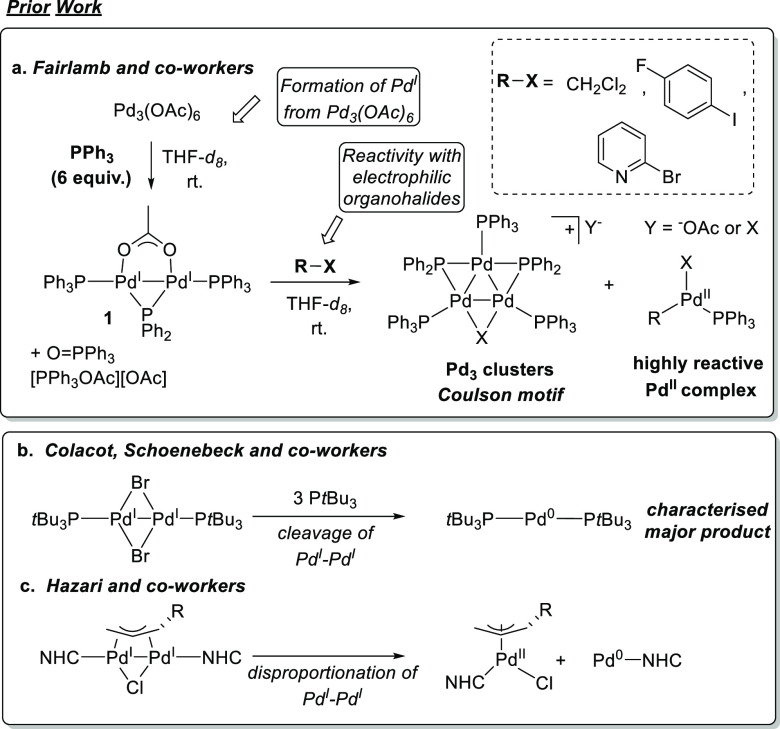
(a) Formation of [Pd^I^_2_(μ-PPh_2_)(μ_2_-OAc)(PPh_3_)_2_] complex **1** and reactivity with organohalides. (b, c) Examples of reactivity of Pd^I^–Pd^I^ dinuclear complexes.

The reactivity of another Pd^I^ dinuclear complex, [Pd(μ-Br)(P^*t*^Bu_3_)]_2_, toward 3 equiv of P*t*Bu_3_ was reported by Colacot and Schoenebeck et al., which afforded [Pd^0^(P*t*Bu_3_)_2_] as the predominant product (Figure 1b).^2^

We considered that another species (e.g., Pd^II^) might accompany Pd^0^ in this type of reaction, at least transiently, as the apparent process is of a disproportionative nature. Indeed, controlled catalyst activation of dinuclear Pd^I^ complexes to Pd^0^ complexes by disproportionation has been proposed as a rationale for their exceptional reactivity.^21−23^ Hazari et al. rationalized that the in situ-formed NHC-containing dinuclear complex(μ-allyl)(μ-Cl)Pd_2_(NHC)_2_ was activated for catalysis via disproportion to Pd^II^Cl(η^3^-allyl)(NHC) and [Pd^0^–NHC] (Figure 1c, NHC = N-heterocyclic carbene ligand *I*Pr).^24^ Thus, we continued to pursue uncovering the distinctive reactivity of Pd^I^-dinuclear complexes so that the implications for cross-coupling catalysis can be more broadly understood. Herein, we report that addition of PPh_3_ to [Pd^I^_2_(μ-PPh_2_)(μ_2_-OAc)(PPh_3_)_2_] **1**, induces disproportionation and subsequent conversion to a dinuclear Pd^I^, namely [Pd^I^_2_(μ-PPh_2_){κ_2_-P,O-μ-P(O)Ph_2_}(κ-PPh_3_)_2_], in addition to [Pd^0^(PPh_3_)_3_]. These two different species exhibit distinct reactivity profiles toward 2-bromopyridine in forming either mononuclear Pd species or higher order Pd_3_ cluster species. The portioning between these two types of species is important for the field of cross-coupling catalysis to consider.

## Results and Discussion

### Reactivity of [Pd_2_(μ-PPh_2_)(μ_2_-OAc)(PPh_3_)_2_] with PPh_3_

**Figure 2:**
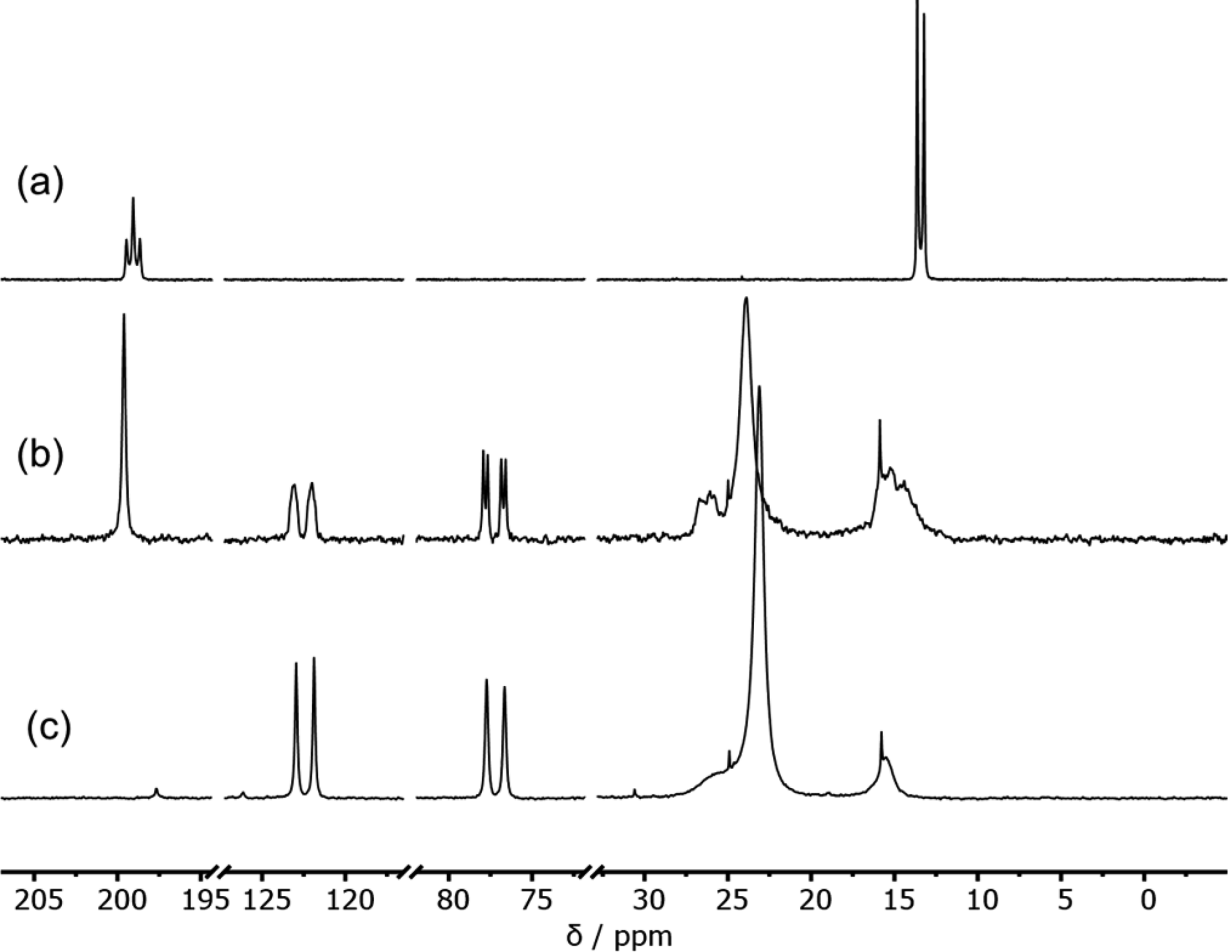
^31^P{^1^H} NMR (THF-*d*_*8*_, 25 °C, 203 MHz) showing reaction of [Pd^I^_2_(μ-PPh_2_)(μ_2_-OAc)(PPh_3_)_2_] **1** with nPPh_3_. (a) Authentic **1**; (b) [Pd^I^_2_(μ-PPh_2_)(μ_2_-OAc)(PPh_3_)_2_] **1** + 1 equiv of PPh_3_ after 0.5 h; (c) [Pd^I^_2_(μ-PPh_2_)(μ_2_-OAc)(PPh_3_)_2_] **1** + 2 equiv of PPh_3_ after 0.5 h. Note: the spectral window has been shortened, as shown by the double diagonal lines on the chemical shift scale line.

In our first reaction, [Pd^I^_2_(μ-PPh_2_)(μ_2_-OAc)(PPh_3_)_2_] **1** was treated with one equivalent of PPh_3_ in THF-*d*_8_ at room temperature. After ca. 30 min, ^31^P NMR spectral analysis revealed the formation of new species (Figure 2b), which could be compared with an authentic sample of **1** (Figure 2a). A phosphorus triplet signal, at δ_P_ 199.6, was assigned as the diphenylphosphido signal of unreacted **1**. A loss of resolution of the triplet was evident, as well as a slight downfield change in chemical shift, δ_P_ 199.1 → 199.6 ppm (Figure 2b). Two new well-resolved resonances appeared at δ_P_ 122.6 and 77.3 ppm, with doublet (*d*) and doublet of doublets (*dd*) multiplicities, respectively, which were found to integrate in a 1:1 ratio (hereafter species **2**; a discussion of coupling constants is found in the text below). A major, broad resonance at δ_P_ 23.9 ppm was evident. Two broad resonances at approximately δ_P_ 26 and 15 ppm were also observed.

Upon reaction of **1** with two equivalents of PPh_3_, further reaction progression was evident by ^31^P NMR spectroscopic analysis after ca. 0.5 h reaction time (Figure 2c). The complete loss of **1** was seen by the disappearance of its phosphido ^31^P NMR resonance. Low-field resonances at δ_P_ 122.6 and 77.3 ppm, which were again present, broaden out, with both appearing as doublets (supporting formation of a new species **2**). The major, broad resonance appeared to shift marginally upfield from δ_P_ 23.9 → 23.1 ppm, which is [Pd^0^(PPh_3_)_3_]. Broadened peaks at ∼ δ_P_ 25.6 and 15.6 ppm were also present, indicating PPh_3_ exchange taking place in the presence of additional phosphine. Treatment of the reaction mixture with 10 equiv of PPh_3_ resulted in disappearance of these broad resonances and migration of the major singlet to δ_P_ 7.6 ppm (externally referenced to H_3_PO_4_(aq) 85% w/w) (see Supporting Information). The migration of this signal upfield (toward the chemical shift of free PPh_3_) is consistent with the reported behavior of [Pd^0^(PPh_3_)_n_] toward additional PPh_3_.^10,16^

**Figure 3:**
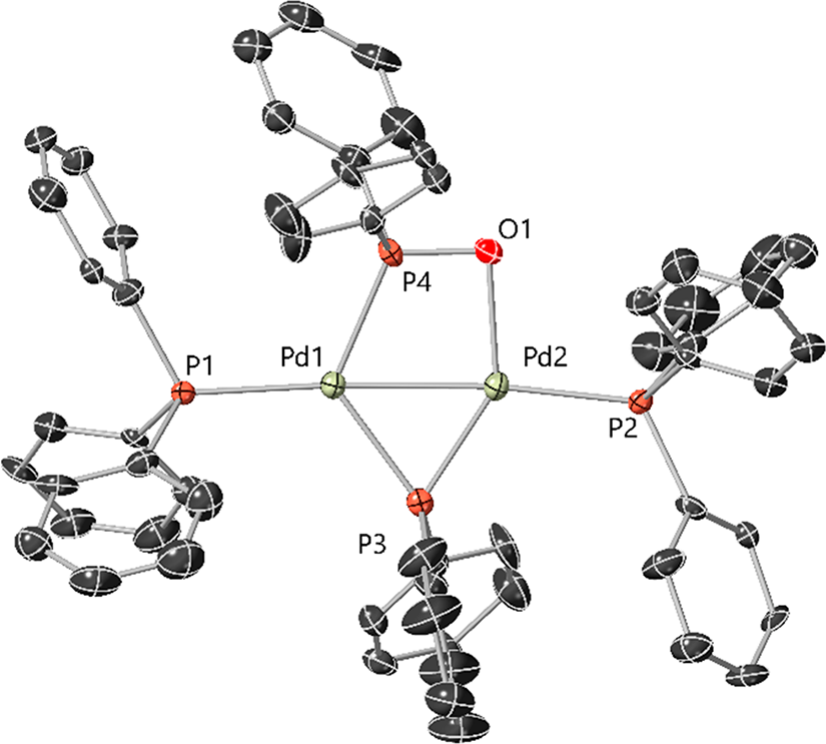
Structure of [Pd_2_(μ-PPh_2_){κ_2_-P,O-μ-P(O)Ph_2_}(κ-PPh_3_)] (2), obtained via X-ray diffraction of a single crystal grown from THF-*d*_*8*_ reaction solution of [Pd^I^_2_(μ-PPh_2_)(μ_2_-OAc)(PPh_3_)_2_] 1 with two equivalents of PPh_3_. Selected interatomic distances /Å: Pd1–Pd2 = 2.5680(10), Pd1–P1 = 2.360(9) Pd2–P2 = 2.275(9), Pd1–P3 = 2.316(3), Pd2–P3 = 2.210(3), P1–P4 = 2.328(3), P4–O1 = 1.550(8), P2–O1 = 2.150(7). Selected interatomic angles /°: Pd1–P3–Pd2 = 69.093, Pd1–Pd2–O1 = 86.162, Pd2–O1–P4 = 93.688. Note: a single molecule from the asymmetric unit cell is shown only for clarity. H-atoms not shown.

A bright red-orange crystal of this new species **2** was grown by carefully layering pentane onto a cooled THF solution of the postreaction mixture of [Pd^I^_2_(μ-PPh_2_)(μ_2_-OAc)(PPh_3_)_2_] **1** and two equivalents of PPh_3_ (stored at −18 °C for ca. 2 days). The crystal was subjected to single-crystal X-ray diffraction analysis which enabled the structural elucidation of this new species to be determined (Figure 3). It is important to note that the asymmetric unit of crystal structural data contains two half complexes of **2**, each of which are disordered about a center of inversion.

The single-crystal X-ray diffraction structure of [Pd_2_(μ-PPh_2_){κ_2_-P,O-μ-P(O)Ph_2_}(κ-PPh_3_)] **2** shows that the P, O, and Pd atoms all lie within approximately the same plane (Figure 3). The Pd atoms are stabilized by bridging μ-phosphido and μ_2_-phosphinito ligands. The Pd1–P4 and the Pd2–O1 bond distances [2.328(3) and 2.150(7) Å, respectively] are significantly less than the sum of the van der Waals radii of the respective atoms, indicating that both O and P atoms of the diphenylphosphinito ligand are bonded to Pd (see DFT calculations later). The Pd1–Pd2 interatomic distance was determined to be 2.5680(10) Å, in-keeping with Pd^I^–Pd^I^ bond lengths of similar Pd^I^ dinuclear complexes.^15,16,25−27^ The P–O bond of the diphenylphosphinito ligand [P4–O1 = 1.550(8) Å] has some double bond-character at 1.479(2) Å),^28^ which falls within the region seen for another bridging diphenylphospinito dinuclear Pd^I^ complex reported by Matt et al.^29^ The structure of **2** is desymmetrized along the Ph_3_P(P3)–Pd(1)–Pd(2)–PPh_3_(P4) bond axis, with the Pd1–P3 interaction being approximately 4% longer than that of P2–P4. It is pertinent to mention that a related PCy_2_-bridged-Pt complex is known.^30a^

**Figure 4:**
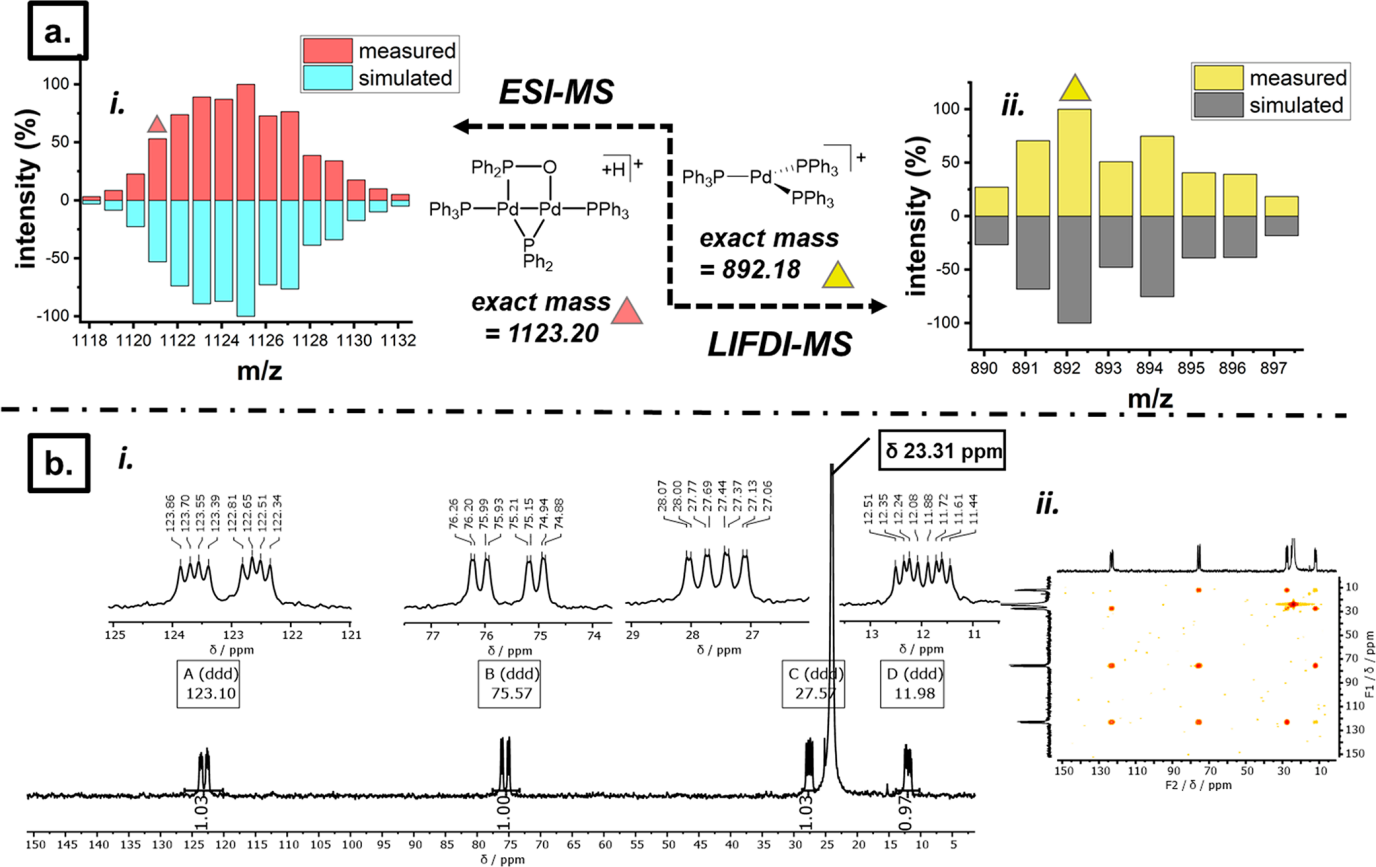
Key characterization data obtained from the postreaction solution (THF-*d*_*8*_), containing the products of the reaction between [Pd^I^_2_(μ-PPh_2_)(μ_2_-OAc)(PPh_3_)_2_] **1** and two equivalents of PPh_3_. (a) Mass spectral data for ions detected in the reaction solution; [Pd_2_(μ-PPh_2_){κ_2_-P,O-μ-P(O)Ph_2_}(PPh_3_)_2_]^+.^, detected by ESI-MS and [Pd^0^(PPh_3_)_3_] detected by LIFDI-MS (right). (b) 1D ^31^P{^1^H} and 2D ^31^P–^31^P{^1^H} COSY NMR spectra (202.5 MHz, CD_2_Cl_2_), recorded at 173 K of the postreaction solution.

Mass spectral analysis (+ve mode) of the postreaction solution provided further evidence for the species formed under the reaction conditions. ESI- and LIFDI-MS analysis were complementary in confirming the presence of [Pd_2_(μ-PPh_2_){κ_2_-P,O-μ-P(O)Ph_2_}(κ-PPh_3_)] **2** as the pseudomolecular ion [M + H]^+^ (exact mass *m*/*z* = 1123.20) (Figure 4a, i) and [M]^.+^ (exact mass *m*/*z* = 1122.09), respectively, with the correct isotopic distribution}. LIFDI-MS analysis indicated the presence of an ion at *m*/*z* = 982.18; this is equal to the exact mass of [Pd^0^(PPh_3_)_3_], detected as [Pd(PPh_3_)_3_]^.+^ (i.e., the radical cation), with the correct isotopic distribution (Figure 4a, i). A low-temperature ^31^P{^1^H} NMR spectrum of a sample from the reaction of **1** with two equivalents of PPh_3_ (173 K, −100 °C, THF-*d*_*8*_, 203 MHz) was recorded (Figure 4b, i). Two low-field resonances at δ_P_ 123.1 and 75.6 ppm (observed as doublets at 298 K) and two new, high-field PPh_3_-type resonances at δ_P_ 27.6 and 12.0 ppm were resolved (previously broad at 298 K), revealing fine coupling between four peaks, each resolving as *ddd* peak multiplicities, giving information important for the complete assignment of the solution structure of **2**. Each phosphorus resonance integrates in a 1:1:1:1 ratio. The P–P spin coupling constants (in Hz) for each resonance are collated in Table 1. The four ^31^P resonances were assigned to the four phosphorus atoms as present in the single-crystal X-ray diffraction structure (Figure 3; **2**,Table 1). A large ^2^*J*_PP_ coupling between the two low-field resonances **A** and **B** (217 Hz) is consistent with the *trans*-arrangement of the phosphinito and phosphide ligands spanning the Pd^I^–Pd^I^ fragment. Likewise, the large ^3^*J*_PP_ coupling constant observed between high-field phosphine-type resonances (131.5 Hz, **C** and **D**) is consistent with a *trans*-configuration.

**Table 1:**
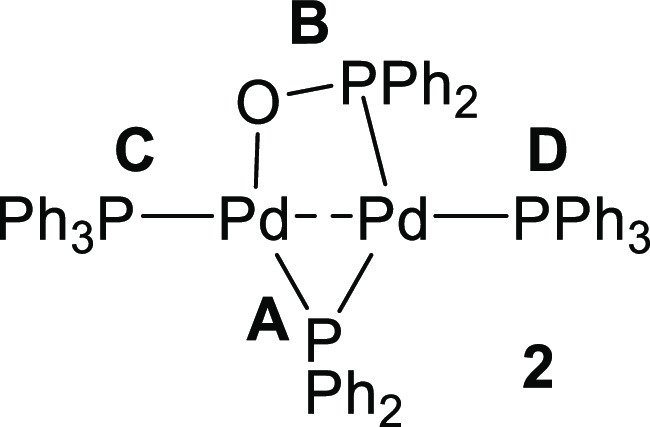
*J*_PP_ Coupling Constants (in Hz) for each ^31^P{^1^H} Resonance in **2**, Recorded at Low Temperature (202.5 MHz, THF-*d*_*8*_, 173 K)

	δ/ppm			
	123.1	75.6	27.6	12.0
label	**A**	**B**	**C**	**D**
**A**	0	217.2	61.5	33.6
**B**	217.2	0	15.8	55.8
**C**	61.5	15.8	0	131.5
**D**	33.6	55.8	131.5	0

The smallest ^3^*J*_PP_ coupling constant observed (15.8 Hz, between **B** and **C**) fits with a *cis*-configuration, coupling through oxygen, between the phosphinito resonance (**B**) and one of the adjacent phosphines (**C**). A larger ^2^*J*_PP_ coupling constant (55.8 Hz) was measured between the phosphido resonance (**B**) and the adjacent phosphine (**D**). The complete connectivity of the phosphorus atoms was confirmed by ^31^P–^31^P COSY-NMR spectral analysis (Figure 4). Hence, the ^31^P NMR spectrum of [Pd_2_(μ-PPh_2_){κ_2_-P,O-μ-P(O)Ph_2_}(κ-PPh_3_)] **2** in THF-*d*_*8*_ agrees with the connectivity suggested by the single-crystal X-ray diffraction structure (Figure 3), along with the cation detected by MS analysis, providing strong evidence that the structure present in THF-*d*_*8*_ solution is **2**, as in the solid-state. In addition to **2**, the presence of [Pd^0^(PPh_3_)_3_] is supported by its detection (radical cation) by LIFDI-MS analysis of the reaction solution.

An additional piece of the jigsaw enabling full reaction mapping was provided by analysis of the ^1^H and ^13^C NMR spectra of the reaction mixtures (500, 125.8 MHz, respectively, in THF-*d*_*8*_). For example, resonances at δ_H_ 2.15 ppm and δ_C_ 166.1 and 20.9 ppm suggested formation of acetic anhydride (confirmed by comparison with an authentic sample). Integration of the ^1^H NMR spectrum confirmed the ratio of [Pd_2_(μ-PPh_2_){κ_2_-P,O-μ-P(O)Ph_2_}(κ-PPh_3_)] **2**:Ac_2_O to be 1:1 (see Supporting Information).

**Figure 5:**
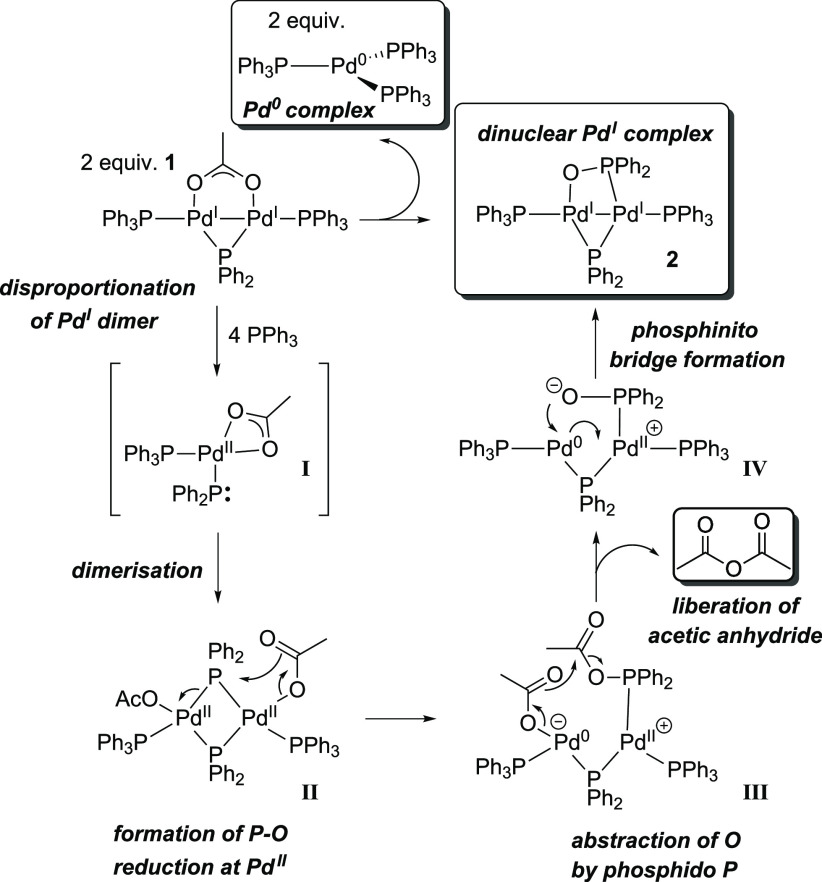
Proposed mechanism for the disproportionation reaction leading to formation of [Pd^I^_2_(μ-PPh_2_){κ_2_-P,O-μ-P(O)Ph_2_}(κ-PPh_3_)_2_] (**2**) and [Pd^0^(PPh_3_)_3_].

Taken together, these data indicate that the room temperature reaction between [Pd^I^_2_(μ-PPh_2_)(μ_2_-OAc)(PPh_3_)_2_] **1** and two equivalents of PPh_3_ cleanly affords [Pd_2_(μ-PPh_2_){κ_2_-P,O-μ-P(O)Ph_2_}(κ-PPh_3_)] **2** and [Pd^0^(PPh_3_)_3_], as evidenced by ^31^P NMR and MS analysis, along with acetic anhydride. A mechanistic scheme describing how the events leading to the formation of **2**, [Pd^0^(PPh_3_)_3_], and Ac_2_O have occurred is shown in Figure 5. The presence of acetic anhydride and **2**, as the other products of this type of process, gives clues as to what has happened to the resulting putative Pd^II^ fragment **I** that formed during disproportionation of the starting dinuclear Pd^I^ complex **1** (Figure 5). We propose that acetic anhydride forms by acyl transfer to acetate at Pd, with loss of one oxygen atom to phosphorus, in forming the phosphinito ligand. In this system, under rigorous Schlenk conditions, acetic anhydride can only derive from the acetate ligands of **2**.

It is important to acknowledge that a dinuclear Pt^II^ complex containing a P,O-bridging phosphinito ligand was formed from the reaction of a higher oxidation state dinuclear Pt^III^ species with hydroxide anion, involving nucleophilic attack of hydroxide at the electrophilic Pt^III^ center.^30b^ Under our conditions, hydroxide anion is not generated (we would expect Ac_2_O to be converted to acetic acid, which was not observed).

The transient stability of Pd^II^ fragment **I** could be conferred by the acetate ligand adopting an *η*_*2*_-OAc binding mode;^31^ however, dimerization could then occur rapidly via bridging interactions from nucleophilic κ_2_-diphenylphosphido ligands,^32^ affording dinuclear Pd^II^ complex **II**. The proximal relationship of the acetate and phosphide ligands sets-up a favored P–O bonding interaction leading to a formal reduction at Pd from two to zero. Subsequent nucleophilic attack on the carbonyl group linked to the phosphorus(V) center in **III** leads to the generation of acetic anhydride, leaving one oxygen atom connected to phosphorus in **IV**, enabling a bridging coordination mode of O to Pd. Thus, the characterized [Pd^I^_2_(μ-PPh_2_){κ_2_-P,O-μ-P(O)Ph_2_}(κ-PPh_3_)_2_] complex **2** is then formed. The redox process is driven by the formation of a strong P–O bond, much like in the formation of Pd^0^ from phosphine-ligated Pd^II^ acetate complexes (vide supra).^10,12^ The reaction of a Pd^I^ dimer by disproportionation, followed by a reduction at Pd is not unprecedented. Disproportionation followed by reduction at the Pd^II^ fragment has been observed by Figueroa et al., but in that case, full reduction of an isopropoxide-bridged, bulky isocyanide-stabilized Pd^I^ dinuclear complex to Pd^0^ occurred, ultimately leading to the formation of a Pd^0^ trimer. Interestingly, acetone and propene were observed by Figueroa et al. as oxidized biproducts, and the Pd^0^ fragments combined to form a Pd_3_ cluster complex.^33^

**Figure 6:**
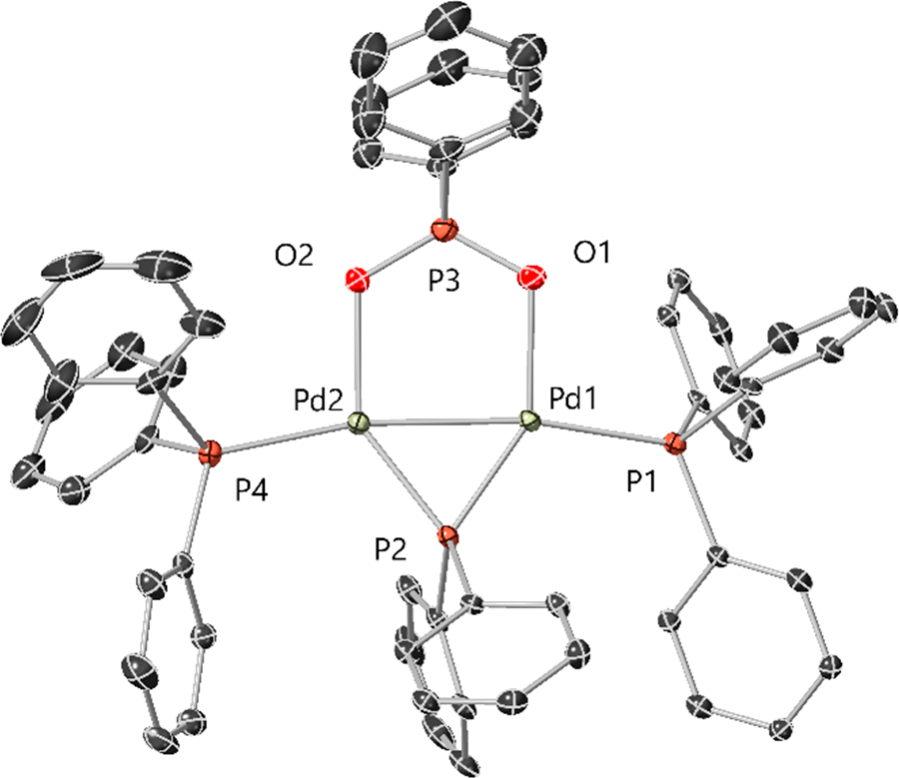
Structure of [Pd_2_(μ-PPh_2_){κ_2_-O,O-μ-P(O)_2_Ph_2_}(κ-PPh_3_)_2_] (**3**) obtained via a X-ray diffraction of a single crystal grown from THF-*d*_*8*_ reaction solution of 1 with 2 equiv of PPh_3_. Selected interatomic distances /Å Pd1–Pd2 = 2.6140(5) P1–Pd1 = 2.2901(12), Pd2–P4 = 2.3095(12), P2–Pd1 = 2.1810(12), P2–Pd2 = 2.1888(12), Pd1–O1 = 2.162(4), Pd2–O2 = 2.186(4), O1–P3 = 1.507(4), O2–P3 = 1.529(4). Selected interatomic angles /° O1–P3–O2 = 120.7(2), Pd1–P2–Pd2 = 73.48(4). H-atoms not shown.

In subsequent experiments, we found that a minor new species crystallized from a reaction mixture where **2** was the major species (grown by layering a THF postreaction solution with hexane, which was stored under Ar at −18 °C). A single crystal was subjected to X-ray diffraction analysis, showing it to be yet another novel Pd^I^ dinuclear complex (**3**) (Figure 6). Analogously to **1** and **2** this complex is bridged by a single μ-diphenylphosphido ligand; however, the secondary bridging ligand is a diphenylphosphinato ligand, which bridges through a κ_2_-O_2_-μ_2_ interaction from the P(O_2_)PPh_2_ moiety. This complex appears to be a rare example, where a phosphinato-type ligand is coordinated to Pd via a bridging interaction through two oxygen atoms. Moissev et al. observed the formation of diphenylphosphinato-bridged Pd^II^ complex, formed from the reaction of [Pd^II^(μ_2_-OAc)(κ-OAc)(PPh_3_)]_2_ with molecular hydrogen in the presence of formic acid.^34^ We tentatively propose the presence of **3** as a minor product to be the result of the oxidation of **2** by trace air during the crystallization, a process that is independent of the formation of **2** (Figure 5).

### Computational Studies for Complex **2**

**Figure 7:**
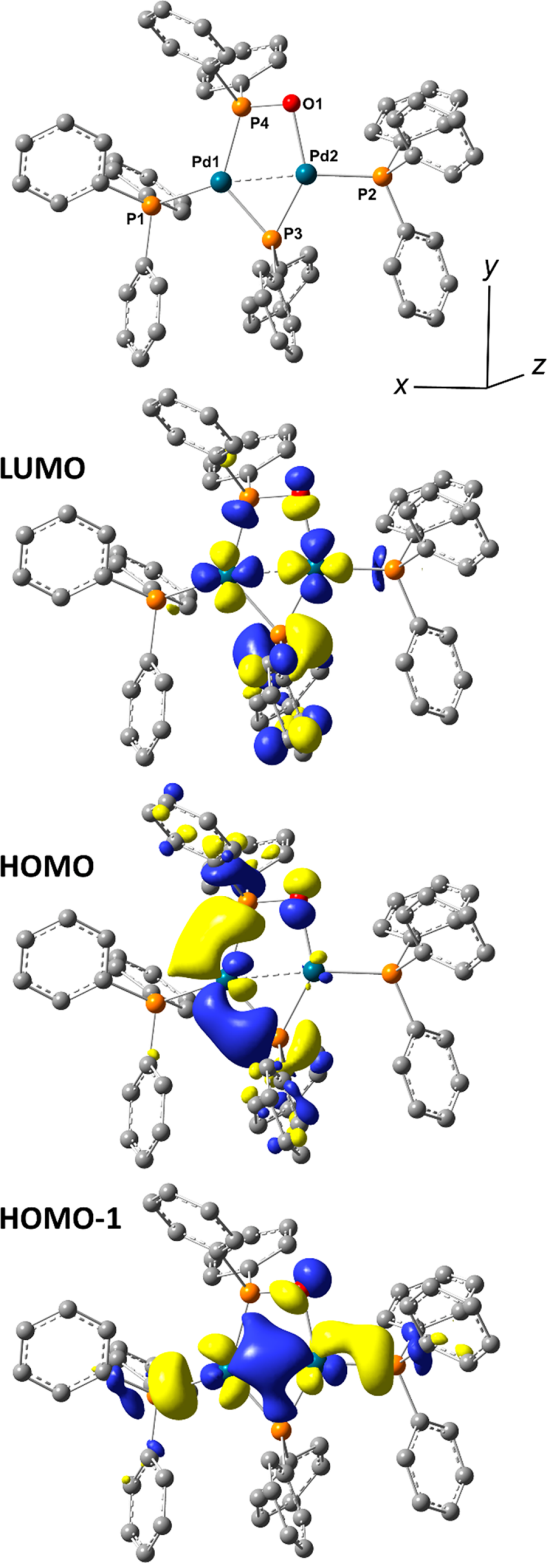
DFT calculated structure of complex **2**, showing the HOMO-1 (P1-Pd1-Pd2-O1-P2 based), HOMO (P3-Pd1-P4-O1 based) and LUMO frontier molecular orbitals.

Computational studies using density functional theory (DFT) calculations for [Pd^I^_2_(μ-PPh_2_){κ_2_-P,O-μ-P(O)Ph_2_}(κ-PPh_3_)_2_] **2** were conducted using the B3LYP/DEF2SVP level of theory with an implicit solvent model (SMD, THF implicit solvent) and empirical dispersion corrections (GD3-BJ) (Figure 7). The calculations reveal a short Pd–Pd bond (2.6046 Å), supporting its diamagnetic properties. The HOMO resides on the “Pd–P–O” moiety, whereas the HOMO-1 resides primarily on the Pd–Pd centers. The LUMO can be found over the phosphide and Pd–Pd centers. The HOMO and HOMO-1 provide clues about the underlying reactivity of **2** toward other electrophilic species, highlighting that oxidation of the phosphinito to phosphinato being clearly feasible, but also direct reaction of **2** with organohalides (presumably involving HOMO-1), similar to what we revealed for dinuclear Pd complex **1**.^12^ Natural bond order (NBO) analysis and calculated Wiberg Indices on complex **2** reveal that there is a reasonable Pd1–Pd2 bonding interaction (see Supporting Information). Wiberg indices for Pd1 and Pd2 were determined to be 0.3167, along with a natural atomic orbital bond order of 0.4737, indicating a partial bond. The Pd2–O1 interaction is relatively weak in comparison to the P4–O1 bond, with Wiberg indices of 0.2697 and 0.9477 respectively. As O1 has a calculated −1 charge, this implies the Pd2–O1 interaction could be more electrostatic than covalent in nature.

### Reactivity of the System with 2-Bromopyridine

We next assessed the reactivity and nucleophilicity of both Pd species – [Pd^I^_2_(μ-PPh_2_){κ_2_-P,O-μ-P(O)Ph_2_}(κ-PPh_3_)_2_] **2** and [Pd^0^(PPh_3_)_3_] (generated by reaction of two equivalents of PPh_3_ with **1**) toward an organohalide, namely 2-bromopyridine. The reason for selecting 2-bromopyridine was that our earlier work^16^ had highlighted the challenges associated with characterizing the product(s) of the reaction mixtures of the Pd species between more typical organohalides (e.g., iodobenzene). Furthermore, employing 2-bromopyridine (having a heteroatom) is arguably like substrates that are more typically used by the synthetic chemistry community.

**Figure 8:**
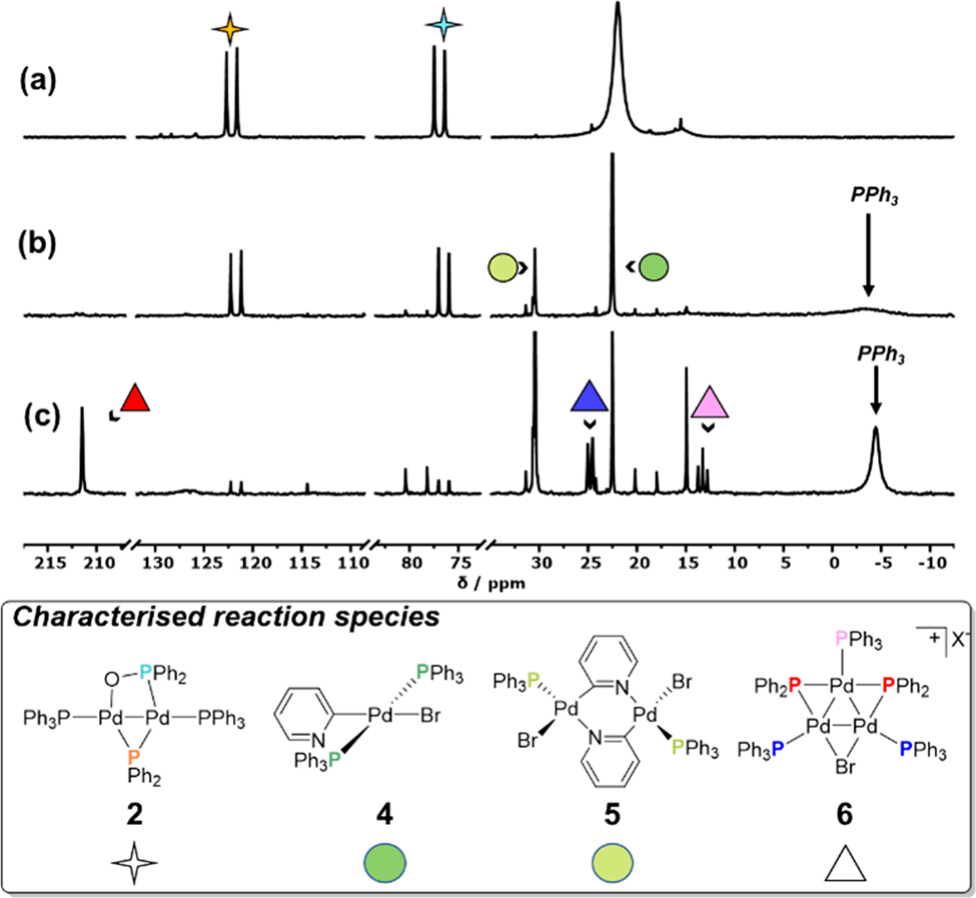
^31^P{^1^H} NMR data (THF-*d*_*8*_, 25 °C, 203 MHz), recorded as a function of time, for the reaction between the postreaction mixture, generated from **1** and PPh_3_, and subsequent reaction with 2-bromopyridine. (a) before, (b) 20 min after, and (c) 13 h after addition of 2-bromopyridine.

A postreaction mixture of Pd^I^_2_(μ-PPh_2_){κ_2_-P,O-μ-P(O)Ph_2_}(κ-PPh_3_)_2_] **2** and [Pd^0^(PPh_3_)_3_] was thus treated with an excess of 2-bromopyridine in THF 25 °C. Over a period of 13 h, ^31^P NMR spectroscopic analysis showed the evolution of several new phosphorus-containing species (Figure 8). Structural assignment was possible for some of the ^31^P-containing reaction products evolving under the reaction conditions. After 20 min ( Figure 8b), the broad signal at δ_P_ 22.0 ppm, [Pd^0^(PPh_3_)_3_], was lost with new resonances forming at δ_P_ 22.5 and 30.5 ppm and a broad peak at ca. δ_P_ – 4.4 ppm, which was assigned to liberated PPh_3_.

The known reaction between [Pd^0^(PPh_3_)_4_] and 2-bromopyridine was carried out,^12^ which allowed for confirmation of the identity of the resonances at δ 22.5 and 30.5 ppm (see Supporting Information for full details). These resonances are associated with *trans*-[Pd^II^(Br)(N,C_2_-pyridyl)(PPh_3_)_2_] and *trans*-[Pd^II^(Br)(N,C_2_-pyridyl)(PPh_3_)]_2_, respectively (**4** and **5**); the oxidative addition complexes of 2-bromopyridine to [Pd^0^(PPh_3_)_2_], derived from [Pd^0^(PPh_3_)_3_],^35^ releasing PPh_3_ (as observed) in less than 10 min.^36^ ESI-MS analysis confirmed the presence of complexes **4** and **5** in the postreaction solution on the basis of observation of the respective *pseudo*-molecular cations [M–Br]^+^.

Unexpectedly, after the addition of 2-bromopyridine, complex **2** was seen to undergo a slower reaction, forming [Pd_3_(μ-Br)(μ-PPh_2_)(PPh_3_)_3_]X **6**, with X most likely being diphenylphosphinite [PPh_2_O]^−^ or Br^–^; the [Pd_3_(μ-Br)(μ-PPh_2_)(PPh_3_)_3_]^+^ species was evident by ^31^P NMR and ESI-MS analysis (Figure 8c). Formation of this particular trinuclear Pd cluster relates to the reactivity of **1** towards 2-bromopyridine, indicating [Pd_3_(μ-X)(μ-PPh_2_)(PPh_3_)_3_]X species as a thermodynamic sink promoted by the bromide nucleofuge.^16^ We noted that the Ac_2_O generated in the preceding reaction was left unreacted, while **4**, **5**, and **6** were formed (comparing ^1^H and ^31^P NMR analysis).

**Figure 9:**
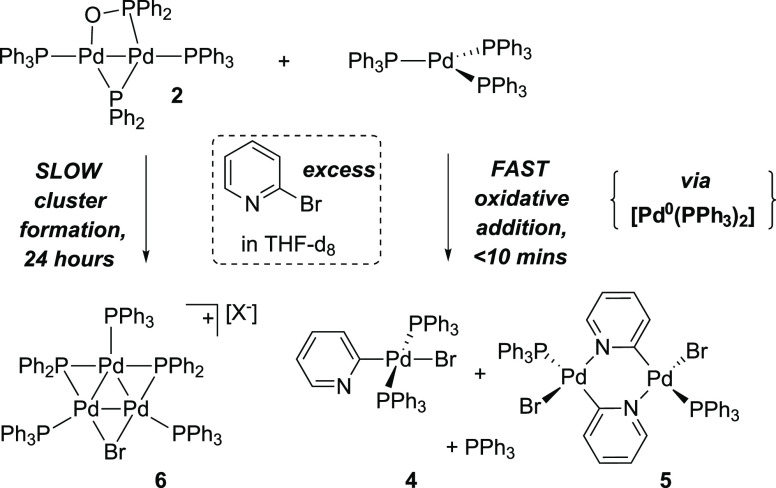
Reactivity of the mixture of Pd^I^ phosphinito complex **2** and [Pd^0^(PPh_3_)_3_]with 2-bromopyridine.

These findings allow us to fully delineate the reaction pathways for **1**, on reaction with two equivalents of PPh_3_, and furthermore 2-bromopyridine, our chosen organohalide to exemplify the link to cross-coupling chemistry (Figure 9).

In conclusion, reaction of [Pd^I^_2_(μ-PPh_2_)(μ_2_-OAc)(PPh_3_)_2_] **1** with two equivalents of PPh_3_ led to disproportionation to [Pd^0^(PPh_3_)_3_] and a new complex [Pd^I^_2_(μ-PPh_2_){κ_2_-P,O-μ-P(O)Ph_2_}(κ-PPh_3_)_2_] **2**. The presence of acetic anhydride as a biproduct of the process indicated that the phosphinito ligand had formed by transfer of an oxygen atom via formal acyl transfer to acetate. The crude reaction mixture containing **2** and [Pd^0^(PPh_3_)_3_] reacted with 2-bromopyridine at different rates: (1) slowly giving [Pd_3_(μ-Br)(μ-PPh_2_)(PPh_3_)_3_]X **6** (formed from **2**); (2) rapidly forming typical oxidative addition products **4** and **5** {formed from [Pd^0^(PPh_3_)_3_]}. These findings reveal that the unique reactivity of dinuclear Pd^I^ complexes enables access to different Pd speciation, with arguably important implications for cross-coupling catalysis,^37^ adding to the complexity of the ubiquitous Pd(OAc)_2_/nPPh_3_ precatalyst system.

## Supporting Information Available

Detailed experimental procedures, compound characterization data, and additional supporting data (PDF)
DFT structures (XYZ)

## Supplementary Material

om1c00347_si_001.pdf

om1c00347_si_002.xyz

## Accession Codes

CCDC 2089316 and 1894929 contains the supplementary crystallographic data for this paper. These data can be obtained free of charge via www.ccdc.cam.ac.uk/data_request/cif, or by emailing data_request@ccdc.cam.ac.uk, or by contacting The Cambridge Crystallographic Data Centre, 12 Union Road, Cambridge CB2 1EZ, UK; fax: +44 1223 336033.
