# Supplementary material for: Reactivity of a Dinuclear PdI Complex [Pd2(μ-PPh2)(μ2-OAc)(PPh3)2] with PPh3: Implications for Cross-Coupling Catalysis Using the Ubiquitous Pd(OAc)2/nPPh3 Catalyst System
Source: Organometallics. 2021 Aug 19;40(17):2995–3002. doi: 10.1021/acs.organomet.1c00347 (PMC8441971; doi:10.1021/acs.organomet.1c00347)
Supplement: Supplementary file 1 — om1c00347_si_001.pdf [file om1c00347_si_001.pdf]

Supporting Information for

Reactivity of a dinuclear Pd^I complex $[Pd_2(\mu\text{-}PPh_2)(\mu_2\text{-}OAc)(PPh_3)_2]$ with PPh_3: Implications for cross-coupling catalysis using the ubiquitous $Pd(OAc)_2/nPPh_3$ catalyst system

Neil W. J. Scott,[a] Mark J. Ford,[b] David R. Husbands,[a] Adrian C. Whitwood[a] and Ian J. S. Fairlamb[a*]

[a] Department of Chemistry, University of York, Heslington, York, YO10 5DD, UK

* ian.fairlamb@york.ac.uk

[b] Bayer AG, Alfred-Nobel-Strasse 50, 40789 Monheim, Germany.

Contents

1. General information 2

1.1. Compound acquisition and purification details 2

1.2. Instruments and methods for compound characterisation 3

2. Experimental procedures and details 4

2.1. Reaction of $[Pd_2(\mu_2\text{-}OAc)(\mu\text{-}PPh_2)(PPh_3)_2]$ (1) with $nPPh_3$ 4

2.1.1. Procedure: reaction between complex **1** and one equivalent of PPh_3 4

2.1.2. Procedure: reaction between complex **1** and two Equivalents of PPh_3 4

2.1.3. Reaction of $[Pd_2(\mu\text{-}PPh_2)(\mu_2\text{-}OAc)(PPh_3)_2]$ (**1**) with 10 equivalents of PPh_3 10

2.2. Reaction of $[Pd_2(\mu_2\text{-}OAc)(\mu\text{-}PPh_2)(PPh_3)_2]$ (1), two equivalents of PPh_3 and 2-bromopyridine 11

2.2.1. Reaction of complex **2** and $[Pd^0(PPh_3)_3]$ and 2-bromopyridine 11

2.2.2. Analysis of background reaction of $[Pd^0(PPh_3)_4]$ with 2-bromopyridine. 14

3. X-Ray Diffraction Details 16

3.1. $[Pd_2(\mu\text{-}PPh_2)\{\kappa_2\text{-}P,O\text{-}\mu\text{-}P(O)Ph_2\}(\kappa\text{-}PPh_3)_2]$ (2) 16

3.2. $[Pd_2(\mu\text{-}PPh_2)\{\kappa_2\text{-}O,O\text{-}\mu\text{-}P(O)_2Ph_2\}(\kappa\text{-}PPh_3)_2]$ (3) 18

4.0. Computational studies: collated energies for complex 2 21

4.1. NBO and Wiberg indices for complex (2) 22

5. References 25

1. General information

1.1. Compound acquisition and purification details

$Pd_3(OAc)_6$ (>99% purity) was obtained fro Precious Metals Online (PMO) and used as received. Triphenylphosphine (PPh_3, Fisher) was purified by recrystallization from hot ethanol and dried under high vacuum, before being stored over P_2O_5 for *ca*. 7 days. 2-Bromopyridine (Merck) was degassed by the freeze-pump-thaw method and stored under N_2. THF was dried by refluxing over sodium metal pieces (2 × 8 hours) before being distilled, transferred to an ampoule and subsequently deoxygenated by bubbling with argon for ca. 30 min. THF-d_8 was dried over freshly sliced K metal pieces for 2 days, at room temperature before freeze-pump-thaw degassing and distilling into an ampoule and stored under Ar. Hexane and pentane were dried by refluxing over freshly sliced sodium metal pieces, before distilling and subsequent storage in an ampoule under N_2.

The Pd^{I}-dinuclear complex; $[Pd_2(\mu\text{-}PPh_2)(\mu\text{-}OAc)(PPh_3)_2]$ **1** and authentic $[Pd_3(\mu\text{-}Br)(\mu\text{-}PPh_2)(PPh_3)_3]Br$ were synthesised using previously published literature procedures.[1] $[Pd^0(PPh_3)_4]$ was prepared using a literature procedure, published by Coulson and stored in an Ar-filled glovebox at –30 °C.[2]

All reactions were carried out either using an Ar-atmosphere glovebox or using Schlenk techniques (high vacuum, liquid nitrogen trap on a standard in-house built dual line manifold {vacuum and N_2}), to eliminate atmospheric air or moisture from the reaction systems. NMR-based experiments were carried out in J. Youngs NMR tubes. Unless otherwise stated, all operations were carried out at room temperature, for which 21-23 °C was recorded.

1.2. Instruments and methods for compound characterisation

NMR spectra were obtained in the solvent indicated, using a Bruker AVIIIHD 500 instrument (500 MHz [^{1}H], 470 MHz [^{19}F], 203 MHz [^{31}P] 125 MHz [^{13}C]) or JEOL ECX400 or JEOL ECS400 spectrometer (400 MHz [^{1}H], 101 MHz [^{13}C] and 377 MHz [^{19}F]). ^{1}H and ^{13}C NMR chemical shifts are reported in parts per million (ppm) and were referenced to the residual non-deuterated solvent of the deuterated solvent used: $C\mathit{H}Cl_3$: δ_H = 7.26 and δ_C = 77.16 ($\mathit{C}DCl_3$), CD_2Cl_2 : δ_H = 5.31 ($CD\mathit{H}Cl_2$) and δ_C = 54.0, THF-d_8 δ δ_H = 3.59 ($OC\mathit{H}_2CH_2$), δ_C = 67.57 $O\mathit{C}H_2CH_2$), δ_H = 1.73 ($OCH_2C\mathit{H}_2$) δ_C= 25.37 ($OCH_2\mathit{C}H_2$). Spectra were typically run at a temperature of 298 K (25 ^{o}C). ^{31}P NMR spectra were generally recorded with proton decoupling unless otherwise stated. ^{31}P NMR spectra were typically recorded using 128 scans and a spectral window of 300 ppm (δ_P +250 to –50 ppm). Chemical shifts (δ_P) for ^{31}P resonances were calibrated by externally referencing to an 85% H_3PO_4 in H_2O (w/w) solution. This was practically carried out by inserting a sealed, vacuum dried capillary tube containing 85% H_3PO_4 in H_2O (w/w) into an NMR tube containing the sample of interest, collecting a ^{31}P NMR spectrum and setting the $H_3\mathit{P}O_4$ resonance to 0 ppm.

HRMS ESI-MS spectra were measured using a Bruker Daltronics micrOTOF MS, Agilent series 1200LC with electrospray ionisation (ESI) or on a Thermo LCQ using electrospray ionisation, with <5 ppm error recorded for all HRMS samples. LIFDI mass spectrometry was carried out using an JEOL AccuTOF GCx-plus instrument (JMS-T200GC), fitted with a probe produced by Linden CMS. The probe was equipped with 13 µm emitters on an AccuTOF. Alternatively, LIFDI-MS was carried out using a Waters GCT Premier MS Agilent 7890A GC instrument. Mass spectral data is quoted as the *m/z* ratio along with the relative peak height in brackets (base peak = 100). Mass to charge ratios (*m/z*) are reported in Daltons. High resolution mass spectra (HRMS) are reported with <5 ppm error (ESI and LIFDI). For clarity, LIFDI data are reported for ^{106}Pd, the most abundant natural isotope of Pd: the 'exact mass' is given for this isotope.

X-ray crystallography: Diffraction data were collected at 110 K on an Oxford Diffraction SuperNova diffractometer with Cu-K_α radiation (λ = 1.54184 Å using a EOS CCD camera. The crystal was cooled with an Oxford Instruments Cryojet. Diffractometer control, data collection, initial unit cell determination, frame integration and unit-cell refinement were carried out with CrysAlisPro.[a] Face-indexed absorption corrections were applied using spherical harmonics, implemented in SCALE3 ABSPACK[b] scaling algorithm within CrysAlisPro. OLEX2[c] was used for overall structure solution, refinement and preparation of computer graphics and publication data. Within OLEX2, the algorithm used for structure solution was ShelXT dual-spaced.[d] Refinement by full-matrix least-squares used the SHELXL[e] algorithm

within OLEX2.[c] All non-hydrogen atoms were refined anisotropically. Hydrogen atoms were placed using a “riding model” and included in the refinement at calculated positions. For the single crystal X-ray structures shown in the main paper, CrystalMarker® X (version 10.4.6) was used and images output as appropriate graphics files.

a CrysAlisPro, Oxford Diffraction Ltd.

b Empirical absorption correction using spherical harmonics, implemented in SCALE3 ABSPACK scaling algorithm within CrysAlisPro software, Oxford Diffraction Ltd.

c “Olex2” crystallography software, J. Appl. Cryst., 2009, 42, 339–341.

d SHELXT – Integrated space-group and crystal-structure determination
G. M. Sheldrick, Acta Cryst. 2015, A71, 3-8

e “Crystal structure refinement with SHELXL”
G.M. Sheldrick, Acta Cryst. 2015, C71, 3-8.

2. Experimental procedures and details

2.1. Reaction of [$Pd_2(\mu_2$-OAc)(μ-$PPh_2)(PPh_3)_2$] (1) with nPPh_3

2.1.1. Procedure: reaction between complex 1 and one equivalent of PPh_3

Figure 2(b), main paper. In an Ar-filled glovebox, [$Pd_2(\mu_2$-OAc)(μ-$PPh_2)(PPh_3)_2$] **1** (5.0 ± 0.05 mg, 5.1 μmol) was weighed into a vial and a solution of PPh_3 (1.4 ± 0.05 mg, 5.3 μmol; 1.04 equiv.) in THF-d_8 (0.5 mL) was added dropwise. The entire solution was transferred into a J. Youngs NMR tube. A colour change from dark red to bright red-orange was immediately observed upon shaking the NMR tube at room temperature. The NMR sample was swiftly introduced to an NMR spectrometer for ^{31}P spectroscopic analysis, where data was collected.

2.1.2. Procedure: reaction between complex 1 and two equivalents of PPh_3

Figure 2(c), main paper. In an Ar-filled glovebox, [$Pd_2(\mu_2$-OAc)(μ-$PPh_2)(PPh_3)_2$] **1** (10.0 ± 0.05 mg, 10.2 μmol) was weighed into a vial and a solution of PPh_3 (5.4 ± 0.05 mg, 20.6 μmol; 2.08 equiv.) in THF-d_8 (0.5 mL) was added dropwise the resulting solution was transferred into a J. Youngs NMR tube. A colour change from dark red to bright red-orange was again immediately observed upon shaking the tube at room temperature. This post-reaction solution was sampled for LIFDI- and ESI-mass spectral analysis by removing a liquid sample in an Ar-glovebox and transferring into a crimp-sealed vial (1 mL capacity). For LIFDI-MS-sampling,

ca. 0.2 mL of the THF-d_8 was extracted with a syringe and needle and the vial was quickly sealed under Ar. For ESI-MS-sampling, a small sample (*ca*. 5 µL) of the THF solution was diluted in toluene 1 mL before being injected directly into the mass spectrometer.

Crystals of $[Pd_2(\mu\text{-}PPh_2)\{\kappa_2\text{-}P,O\text{-}\mu\text{-}P(O)Ph_2\}(\kappa\text{-}PPh_3)_2]$ (**2**) were grown from this THF-d_8 solution by layering with pentane (THF-d_8/pentane = 1:4 v/v) and subsequent storage over *ca.* 2 days at -18 ^oC, under an atmosphere of argon. Red crystals formed alongside a yellow precipitate most likely $[Pd^0(PPh_3)_3]$, which was observed as a major solution species. Red crystals were purposely selected and subjected to X-ray crystallographic analysis.

Red crystals of $[Pd_2(\mu\text{-}PPh_2)\{\kappa\text{-}2\text{-}O,O\text{-}\mu\text{-}P(O)_2Ph_2\}(\kappa\text{-}PPh_3)_2]$ (**3**) were grown form a similar solution by layering with dry hexane (THF-d_8/hexane = 1:4 v/v) and subsequent storage over 7 days at –18 ^oC

Bright red crystals, isolated by filtration, from the same batch of crystals from which the X-ray structure of 2 was obtained, were picked, combined manually (using tweezers) under air. These selected crystals were confirmed by XRD to be compound **2** (selected and collected as detailed above). The crystals were subsequently taken into a glovebox and dissolved in THF-d_8 under an atmosphere of argon. A ^{31}P NMR spectrum is shown in Figure S1 (see overleaf).

2.1.2.1. ^{31}P NMR spectrum of XRD verified crystals of complex 2

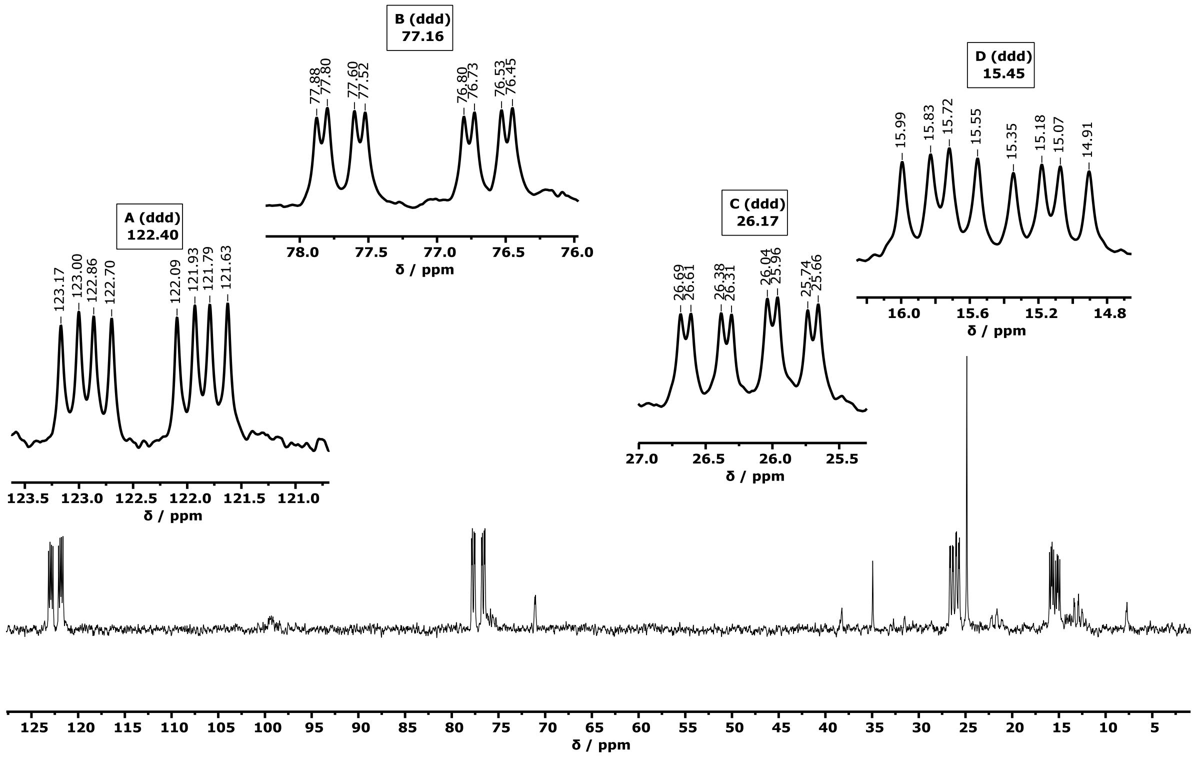

Figure S1 ^{31}P NMR spectrum (203 MHz, 1024 scans, THF-d_8) of crystals of [Pd_2(μ-PPh_2){κ_2-P,O-μ-P(O)Ph_2}(κ-PPh_3)$_2$] (2), as verified by X-ray diffraction analysis.

In the above case, with the handpicked crystals (Figure S1), there was a small amount of contamination with other ^{31}P-containing species – with one species identified as O=PPh_3(δ_P 24.2 ppm), a likely breakdown product of the system on short exposure to air.

2.1.2.2. ^{31}P COSY Spectrum of the Solution Generated from <u>1</u> & two Equivalents of PPh_3 (Figure 4)

Shown in smaller format in Figure 4(ii), main paper.

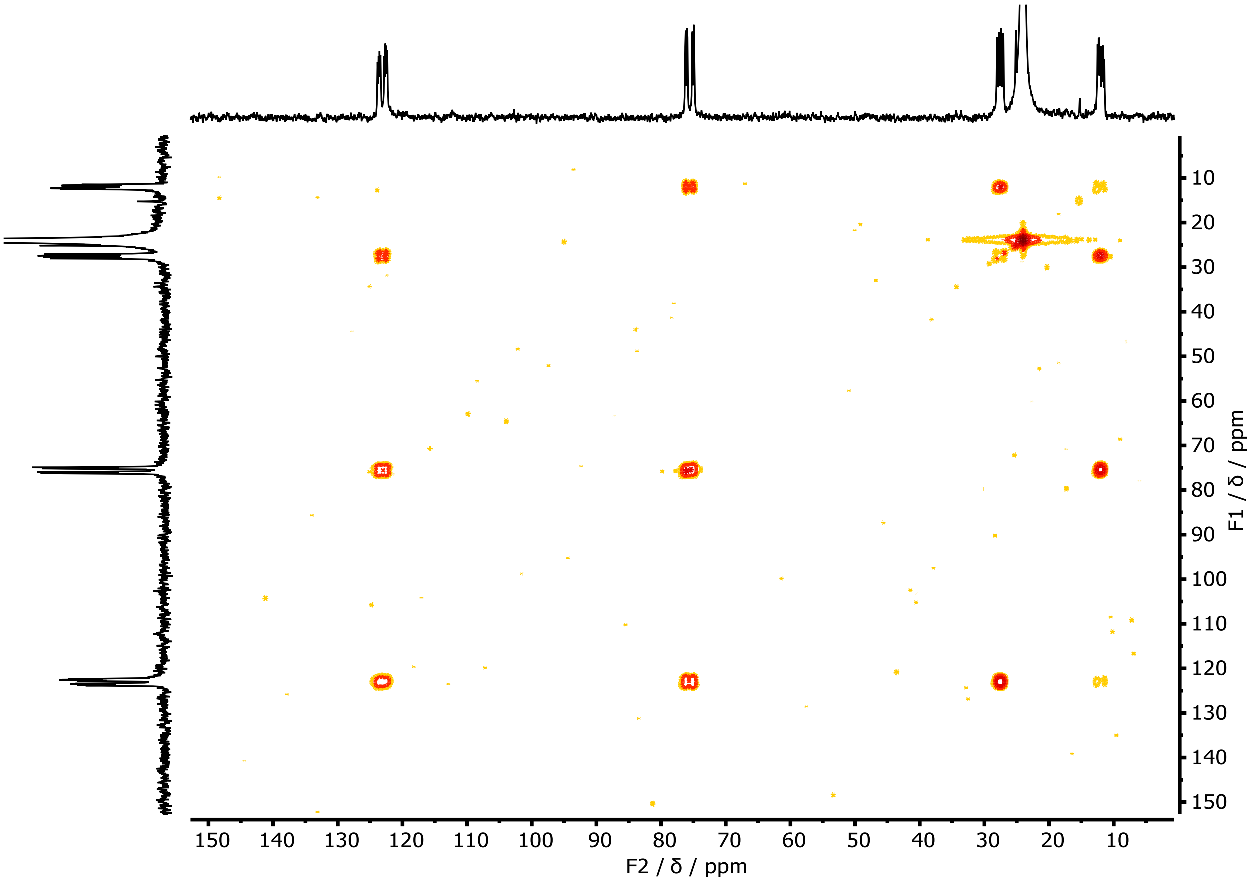

Figure S2 ^{31}P COSY NMR spectrum (202.5 MHz, recorded at 173 K, THF-d_8) of the mixture of 2 and [$Pd^0(PPh_3)_3$], generated from reaction of complex 1 with two equivalents of PPh_3.

2.1.2.3. Evidence for the formation of Ac_2O accompanying complex 2

^1H and ^{13}C NMR spectroscopic evidence was gained for the formation of acetic anhydride (Ac_2O) form the reaction of **1** with two equivalents of PPh_3 (Figure S3). Ac_2O was identified by ^1H NMR analysis as a singlet at δ_H 2.15 ppm (externally referenced to residual ^1H signal of THF-d_8 {3.57 ppm}) and by ^{13}C NMR as two singlets at δ_C 21.99 and 167.21 ppm (externally referenced to ^{13}C signal of THF-d_8 {67.57 ppm}).

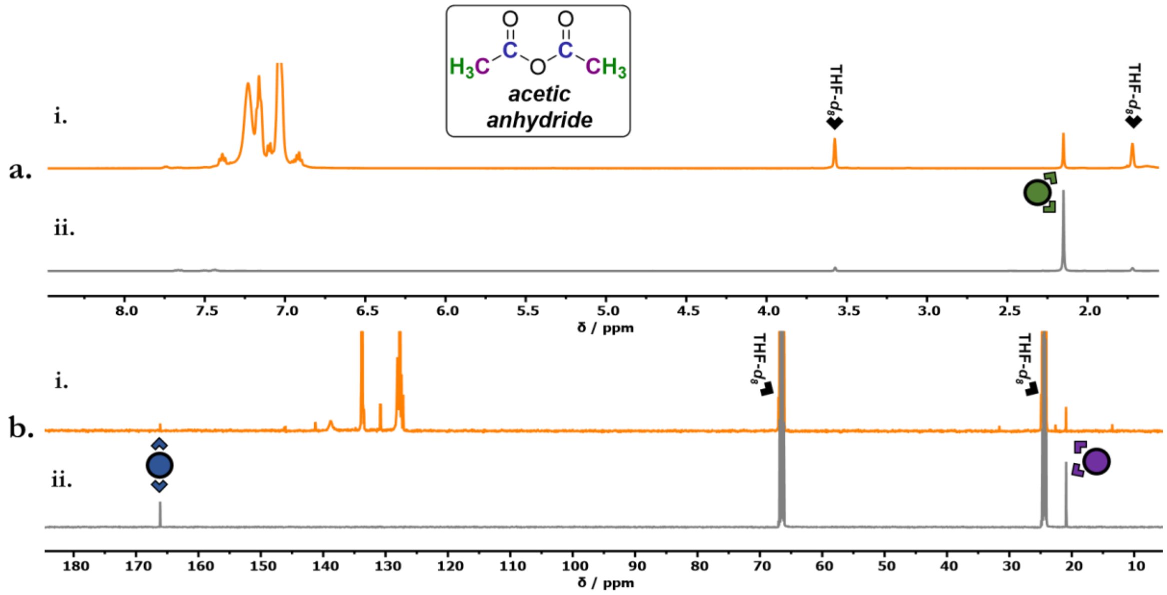

Figure S3 (a.) ^1H NMR (500 MHz) and (b.) ^{13}C NMR (126 MHz) stacks of (i.) crude, post reaction mixture following treatment of complex 1 and two equivalents of PPh_3 and (ii.) a solution containing authentic Ac_2O. All samples run in THF-d_8.

2.1.2.4. Further ^{1}H NMR analysis showing 1:1 ratio of complex 2 with acetic anhydride

Due to overlap of peaks of **2** and [Pd^{0}(PPh$_3$)$_3$] it was challenging to ascertain the comparative stoichiometry with acetic anhydride (Ac$_2$O) from the ^{1}H NMR spectrum of the crude reaction mixture. The peak at δ_H 6.92 ppm has been assigned as PCC**H** of the bridging diphenylphosphido ligand of **2**, due to ^{1}H chemical shift similarities with the bridging diphenylphosphido ligand of **1**.[1] If the Ac$_2$O peak is set as 6H, an integration of 6.44 H is measured for the peak at δ_H 6.92 ppm, which is higher than the 4H expected. This is a result of an overlap with phenyl resonances derived from [Pd^{0}(PPh$_3$)$_3$]. What can be reasoned from this observation is that the amount of Ac$_2$O is, to a close approximation, 1:1. See Figure S4.

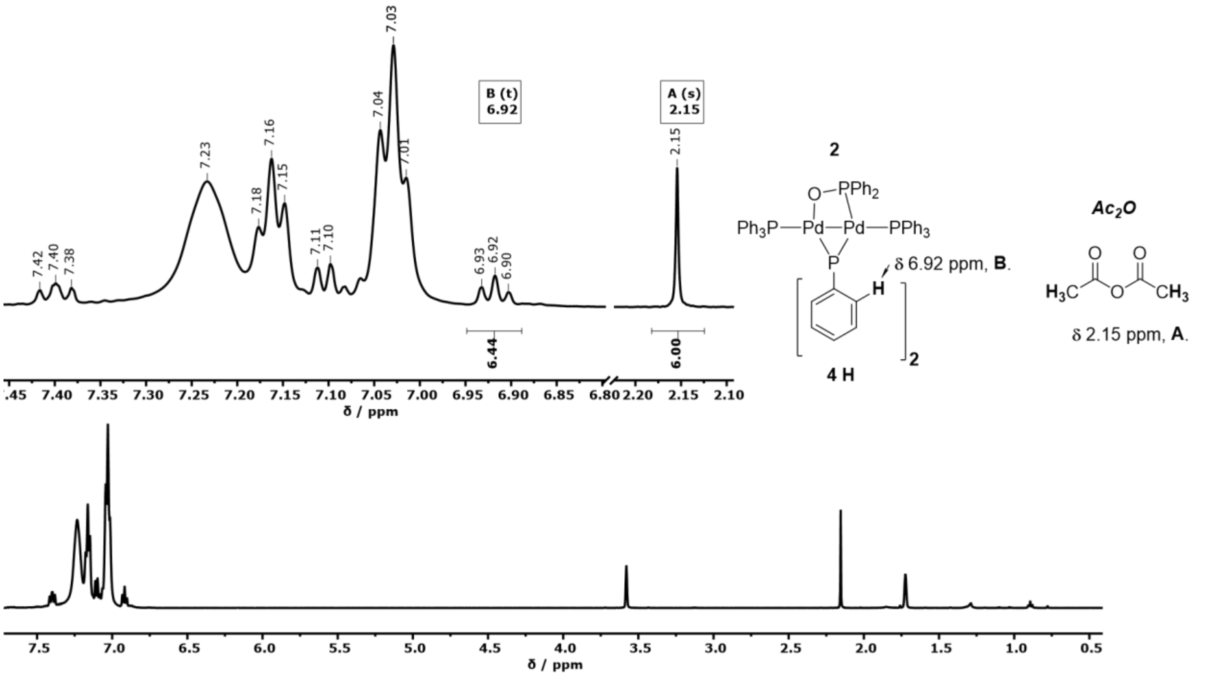

Figure S4 ^{1}H NMR (500 MHz) spectrum of complex 1 / two equivalents of PPh$_3$ post-reaction solution. Expansion of the aromatic region (6.8 – 7.45 ppm) showing acetic anhydride (Ac$_2$O). Integrations of the peak assigned as the four *ortho*-protons of the μ-phosphido ligand of 2 and the Ac$_2$O (C*H*$_3$) peaks were compared.

2.1.3. Reaction of $[Pd_2(\mu\text{-}PPh_2)(\mu_2\text{-}OAc)(PPh_3)_2]$ (1) with 10 equivalents of PPh_3

In an Ar-filled glovebox, complex **1** (5.0 ± 0.05 mg, 5.1 μmol) was weighed into a vial and a solution of PPh_3 (13.4 ± 0.05 mg, 0.050 μmol; 10 equiv.) in THF (0.5 mL) was added dropwise. The solution was transferred to a J. Youngs NMR tube. A colour change from dark red to bright red-orange was again immediately observed upon shaking the NMR tube at room temperature. The reaction was analysed then analysed by ^{31}P NMR spectroscopy (Figure S5).

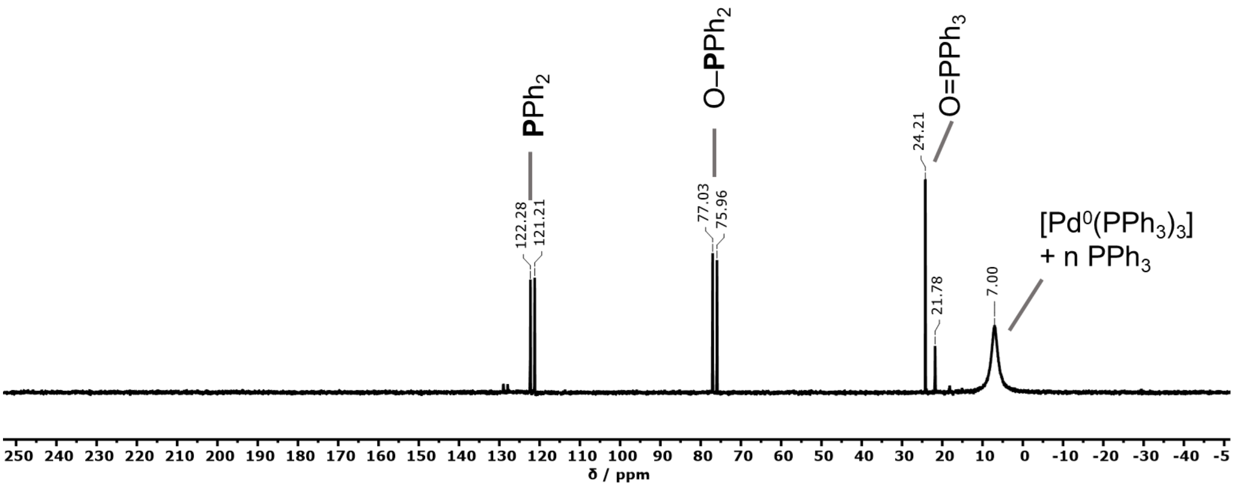

Figure S5 ^{31}P NMR spectrum (203 MHz, 1024 scans, THF, unlocked, (externally referenced to H_3PO_4 (*aq*) 85% {w/w}).

The peak representative of $[Pd^0(PPh_3)_3]$, in exchange with $nPPh_3$ (excess), has migrated upfield, in line with previous observations.[1,3] In addition, a loss of resolution of the PPh_3 resonances of **2** was observed, most likely due to intermolecular exchange of PPh_3 ligands between complex **2**, the Pd^0 complex and its free form.

2.2. Reaction of $[Pd_2(\mu_2\text{-}OAc)(\mu\text{-}PPh_2)(PPh_3)_2]$ (1), with two equivalents of PPh_3 and 2-bromopyridine

2.2.1. Reaction of complex 2 and $[Pd^0(PPh_3)_3]$ and 2-bromopyridine

The reaction between complex **1** and two equivalents of PPh_3 was carried out as above. In an Ar-filled glovebox, the solution was treated with bromopyridine (5 μL, 8.25 mg, 0.052 mmol => 5 equivalents) *via* a microsyringe within 24 hours of generation of the stable solution of complex **2** and $[Pd^0(PPh_3)_3]$. The evolution of the resulting red solution was monitored over the course of 13 hours by ^{31}P NMR spectroscopic analysis. The ^{31}P NMR spectral evidence for the formation of $[Pd_3(\mu\text{-}Br)(\mu\text{-}PPh_2)(PPh_3)_3]X$ (**6**) is given through comparison of the crude reaction solution against an authentic standard of $[Pd_3(\mu\text{-}Br)(\mu\text{-}PPh_2)(PPh_3)_3]Br$, as shown in Figure S6. The $[Pd_3(\mu\text{-}Br)(\mu\text{-}PPh_2)(PPh_3)_3]Br$ authentic standard was synthesized according to an adapted[4-5] method as previously reported.[1]

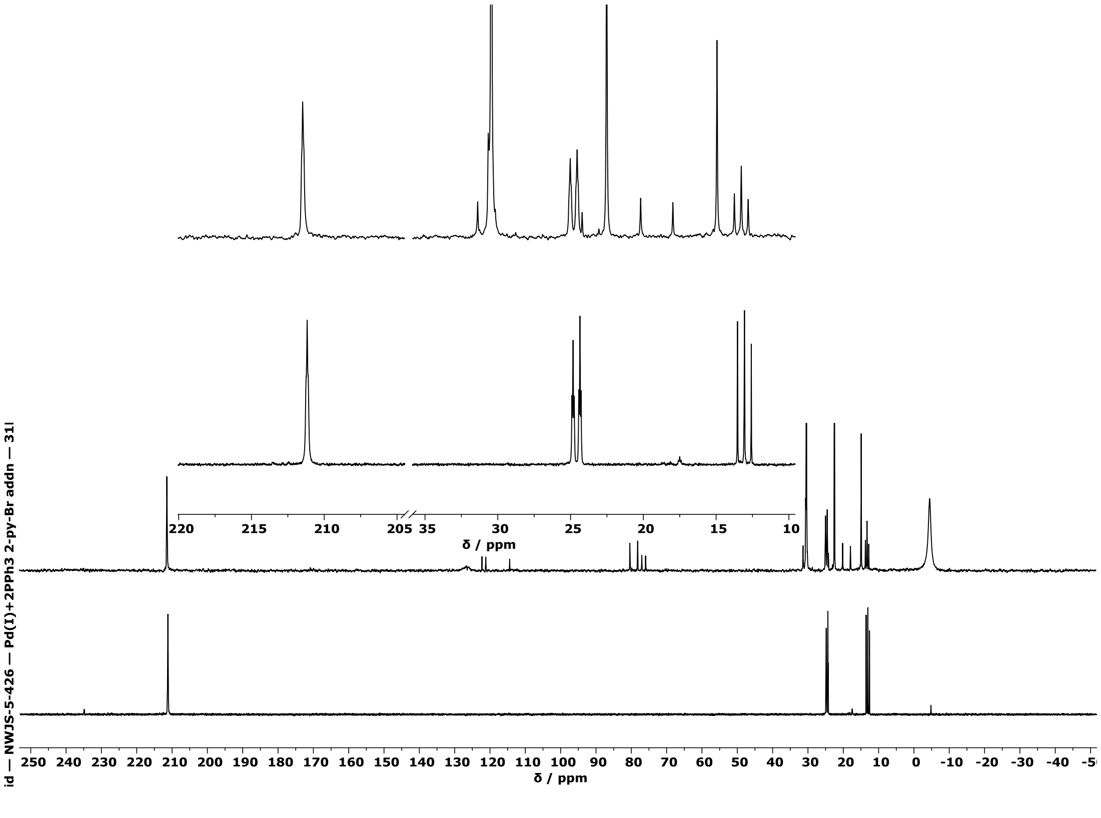

Figure S6 ^{31}P NMR evidence for the formation of $[Pd_3(\mu\text{-}Br)(\mu\text{-}PPh_2)(PPh_3)_3]X$ (6), from the reaction of the solution generated from 1 and two equivalents of PPh_3 with 2-bromopyridine. A stack of ^{31}P NMR spectra from the reaction between 2-bromopyridine (above) and an authentic sample of $[Pd_3(\mu\text{-}Br)(\mu\text{-}PPh_2)(PPh_3)_3]Br$, indicating that that $[Pd_3(\mu\text{-}Br)(\mu\text{-}PPh_2)(PPh_3)_3]Br$ is present in the former solution. *Inset.* An expansion of the same stacked spectra, with excised regions from ~35 to 205 ppm, for clarity.

The presence of the tripalladium cluster cation $[Pd_3(\mu\text{-}Br)(\mu\text{-}PPh_2)(PPh_3)_3]^+$ was evident *via* ESI-mass spectral analysis (Figure S7). Further evidence of the product of the oxidative addition reaction of $[Pd^0(PPh_3)_3]$ with 2-bromopyridine, forming $[Pd^{II}(Br)(N,C^2$-*pyridyl*$)(PPh_3)_2]$ **4** was obtained *via* ESI-MS (detected as the $[M–Br]^+$ ion 708.12 Da {m/z}; [Pd(N, C2*pyridyl*)$(PPh_3)_2]^+$) (Figure S7).

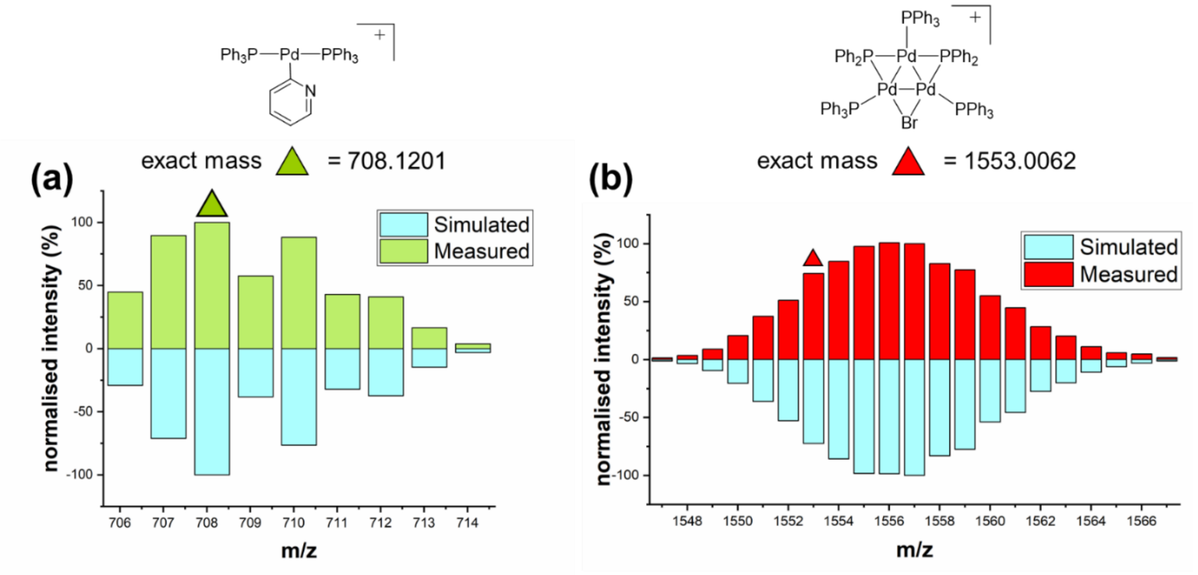

Figure S7 ESI-Mass spectral evidence indicating formation of (a) $[Pd^{II}Br(C^2$-*pyridyl*$)(PPh_3)_2]$ (4): detected as $[M–Br]^+$; $[Pd(C^2\text{-pyridyl})(PPh_3)_2]^+$. (b) $[Pd_3(\mu\text{-}Br)(\mu\text{-}PPh_2)(PPh_3)_3]^+$. Isotopic distributions for the measured *versus* the simulated pattern is given.

Integrated NMR spectra for the reaction mixture in Figure 8 (main paper)

The ^{31}P NMR spectra of the reaction product of **2** with 2-bromopyridine are shown in figures S8 and S9, below.

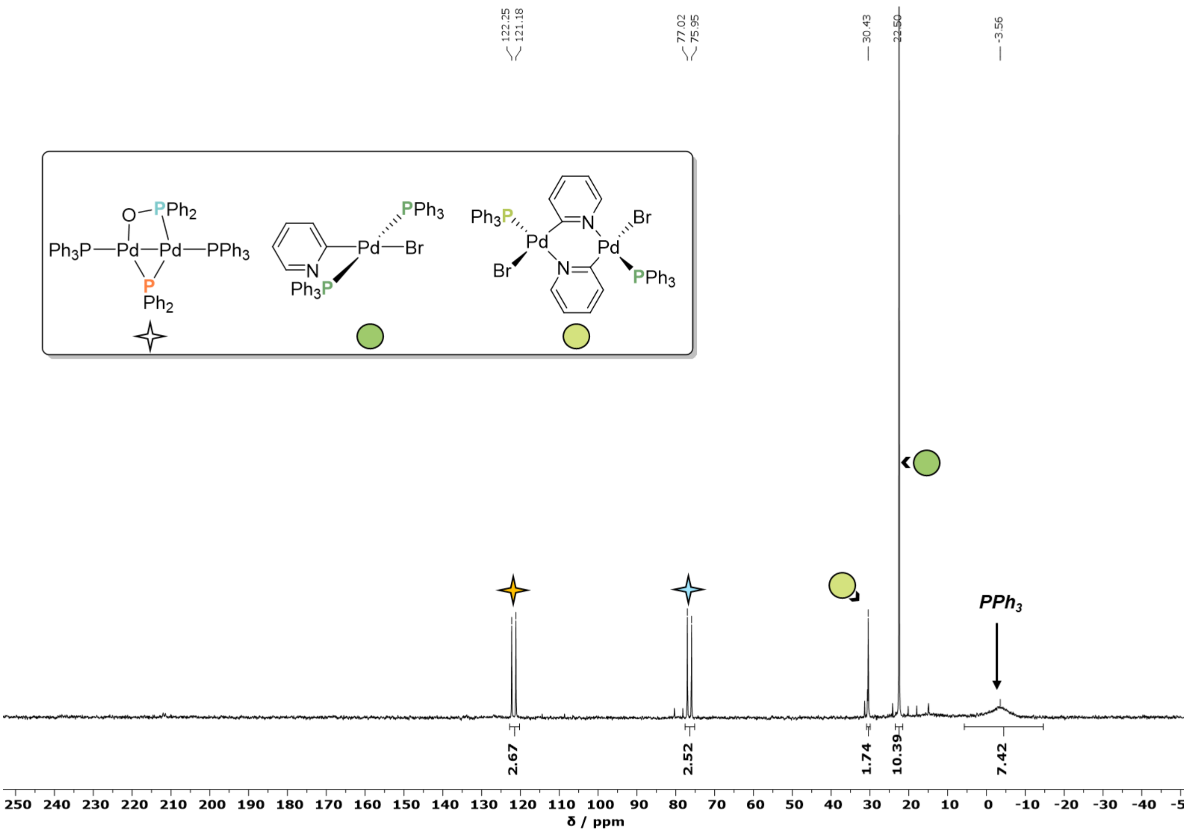

Figure S8 Full spectrum from figure 8 (b) (main paper). Recorded 20 minutes after addition of 2-bromopyridine to the reaction solution. Showing identified species and integration values. $^{31}P\{^{1}H\}$ NMR, 203 MHz, THF-d_8, 25 ^{o}C.

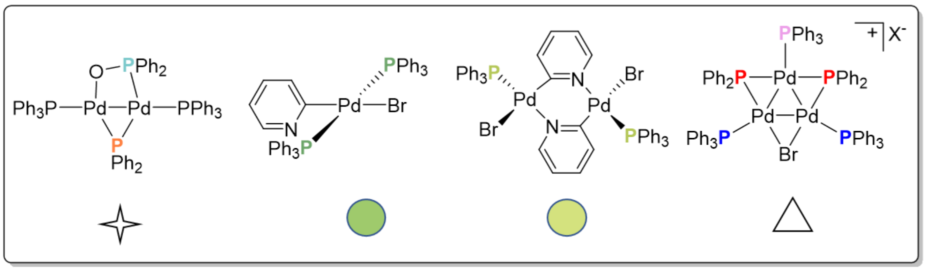

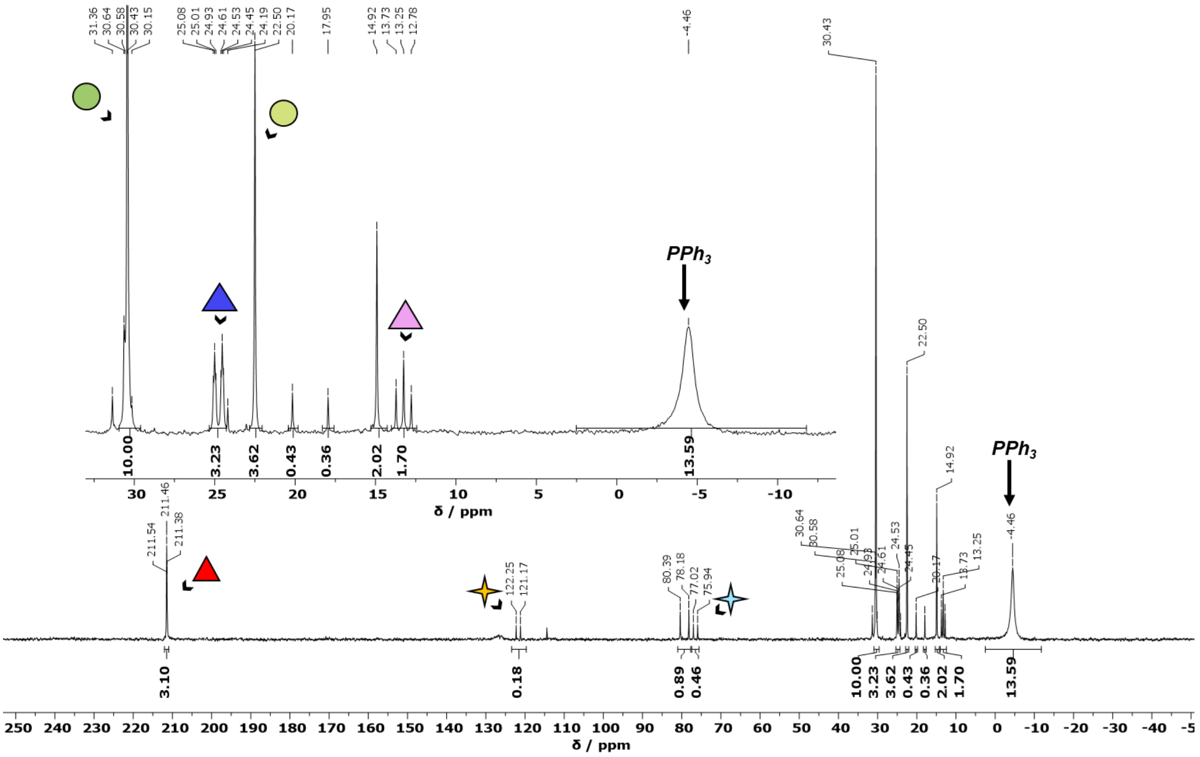

Figure S9 Full spectrum from figure 8 (c) (main paper). Inset: expansion of spectral region between –13 - 33 ppm. Recorded 13 hours minutes after addition of 2-bromopyridine to the reaction solution. Showing identified species and integration values. $^{31}P\{^{1}H\}$ NMR, 203 MHz, THF-d_8, 25 °C.

2.2.2. Analysis of background reaction of $[Pd^0(PPh_3)_4]$ with 2-bromopyridine.

A background reaction enabled confirmation and NMR identification of products of the oxidative addition of $[Pd^0(PPh_3)_3]$ (generated *in situ* by disproportionation of complex **1** after addition of PPh_3). Here, $[Pd^0(PPh_3)_4]$ was used as a proxy for $[Pd^0(PPh_3)_3]$ (in solution, $[Pd^0(PPh_3)_4]$ is known to lose a PPh_3 ligand, readily forming $[Pd^0(PPh_3)_3]$ (as well as, to some extent, $[Pd^0(PPh_3)_2]$).[6]

In an Ar-filled glovebox, $[Pd^0(PPh_3)_4]$ (10 mg, 8.7 μmol) dissolved in 0.5 mL of THF-d_8. 2-bromopyridine (3.0 mg, 19.1 μmol, 2.2 equivalents) was added dropwise *via* a microsyringe.

The mixture was transferred into a J. Youngs NMR tube before being sealed and introduced to an NMR spectrometer for *in operando* ^{31}P spectroscopic analysis (25 °C) (Figure S10). Initially, after *ca*. 20 minutes, two major peaks were identified at δ_P 22.5 and –4.5 ppm, which were assigned as *trans*-[Pd^{II}(Br)(N,C^2-pyridyl)(PPh_3)$_2$] **4** and liberated PPh_3 respectively. An initially minor peak (δ_P 30.5 ppm) was confirmed as the Pd^{II} dinuclear complex *trans*-[Pd^{II}(Br)(N,C^2-pyridyl)(PPh_3)]$_2$ **5**, with reference to literature and an authentic sample.[7] The minor peak, representing **5**, at δ_P 30.5 and that representing PPh_3 grew at expense of the peak representing **4**, eventually becoming a major species over the course of 24 hours. This supported the notion that *trans*-[Pd^{II}(Br)(N,C^2-pyridyl)(PPh_3)$_2$] **4** forms initially as the kinetic oxidative addition product, which dimerises to *trans*-[Pd^{II}(Br)(N,C^2-pyridyl)(PPh_3)]$_2$ **5** with release of PPh_3. The observation that *trans*-[Pd(Br)(N,C^2-pyridyl)(PPh_3)$_2$] **4** converts to [Pd(Br)(N,C^2-*pyridyl*)(PPh_3)]$_2$ **5** over time at 25 °C, with release of free PPh_3 indicates that **5** is more thermodynamically stable than *trans*-[Pd(Br)(N,C^2-pyridyl)(PPh_3)$_2$]. This conversion may be due to increased stability of the dimeric which forms a 6-membered ring, known to adopt a boat conformation.[7] There also may be an entropic contribution to this stability due to the increased solution disorder because of two molecules of monomer complex affording one dinuclear complex releasing two equivalents of PPh_3 for each dimerization reaction. It is notable that the more thermodynamically stable dimeric complex features substitution of dative bonds from the pyridine donor at expense of the PPh_3, the latter of which are known to bond more strongly with Pd^{II} than pyridines.

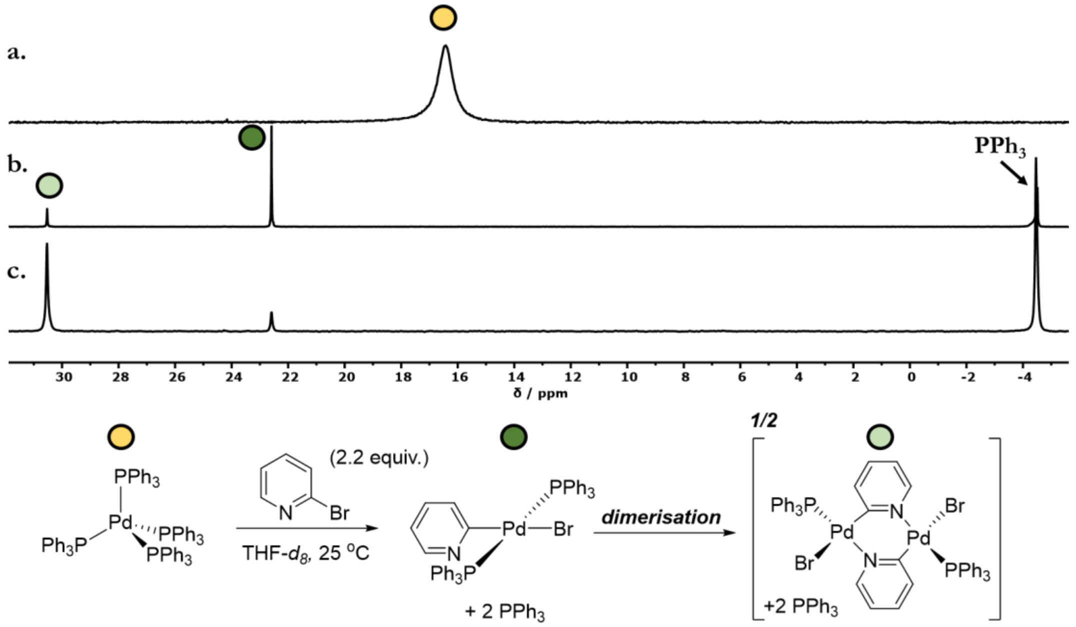

Figure S10 ^{31}P NMR (202.5 MHz, 298 K) tracking of the room temperature reaction between [Pd^0(PPh_3)$_4$] and 2-bromopyridine in THF-*d_8*. (a.) [Pd^0(PPh_3)$_4$] in the absence of 2-bromopyridine. (b.) *ca.* 20 minutes after addition of 2-bromopyridine (2.2 equivalents) (c.) 48 hours after addition of 2-bromopyridine.

3. X-Ray Diffraction Details

3.1. [Pd_2(μ-PPh_2){κ$_2$-P,O-μ-P(O)Ph_2}(κ-PPh_3)$_2$] (2)

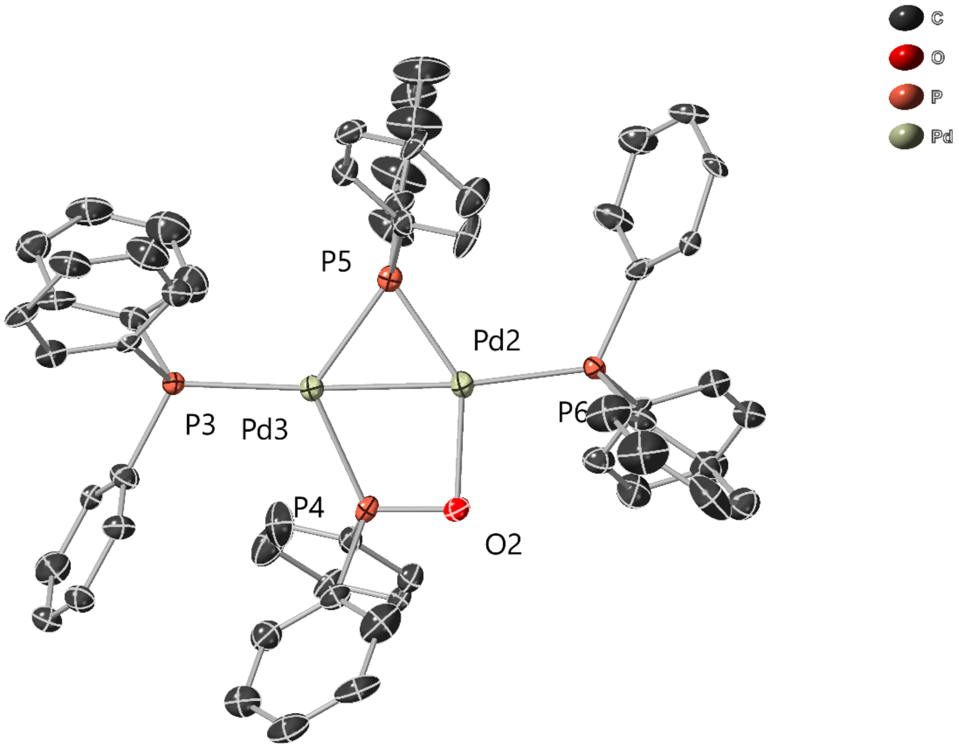

Figure 11 X-ray crystal structure of [Pd_2(μ-PPh_2){κ$_2$-P,O-μ-P(O)Ph_2}(κ-PPh_3)$_2$] (2)

Table S1 X-ray diffraction data for 2

**Empirical formula**	**$C_{60}H_{50}OP_4Pd_2$**
**Formula weight**	1123.68
**Temperature/K**	109.95(10)
**Crystal system**	triclinic
**Space group**	P-1
**a/Å**	9.3916(4)
**b/Å**	12.1359(5)
**c/Å**	23.1782(10)
**α/°**	78.881(4)
**β/°**	79.050(4)
**γ/°**	74.384(4)
**Volume/Å^3**	2469.7(2)

**Z**	2
**ρ_{calc}g/cm^3**	1.511
**μ/mm^{-1}**	7.422
**F(000)**	1140.0
**Crystal size/mm^3**	0.171 × 0.066 × 0.046
**Radiation**	CuKα (λ = 1.54184)
**2Θ range for data collection/°**	7.65 to 134.16
**Index ranges**	-11 ≤ h ≤ 11, -12 ≤ k ≤ 14, -21 ≤ l ≤ 27
**Reflections collected**	16496
**Independent reflections**	8824 [R_{int} = 0.0308, R_{sigma} = 0.0437]
**Data/restraints/parameters**	8824/36/793
**Goodness-of-fit on F^2**	1.228
**Final R indexes [I>=2σ (I)]**	R_1 = 0.0556, wR_2 = 0.1194
**Final R indexes [all data]**	R_1 = 0.0629, wR_2 = 0.1226
**Largest diff. peak/hole / e Å^{-3}**	0.73/-0.65
**CCDC number**	2089316

Refinement Special Details

The asymmetric unit contained two half complexes, each of which were disordered about a center of inversion. The first complex had a common site for one palladium and one terminal phosphorus (Pd1 & P1), the rest of this complex was modelled in two positions with equal occupancy in each site.

The second complex was completely disordered about an inversion center located close to the midpoint between the two palladiums Pd2 & Pd3. Several constraints and restraints were applied to the structure as follows: The ADP of C2A, C2B, C9A, C9B, C43 and C73 were restrained to be approximately isotropic. The ADP of the following pairs of atoms which were close in space were constrained to be equal: C1A & C1B, C2A & C2B, C3A & C3B , C4A & C4B, C5A & C5B, C6A & C6B, C7A & C7B, C9A & C9B, C10A & C10B, C11A & C11B, C13A & C13B, C14A & C14B, C16a & C16B, C18A & C18B, C25A & C25B, C27A & C27B, C28A & C28B, C32 & C84, C37 & C85, C39 & C87, C43 & C73, C44 & C74, C54 & C72, C49 & C67, C51 & C69, C55 & C61.

All the phenyl rings were constrained to be regular hexagons with a C-C bond-length of 1.39 angstroms.

3.2. [Pd_2(μ-PPh_2){κ$_2$-O,O-μ-P(O)$_2$$Ph_2$}(κ-$PPh_3$)$_2$] (3)

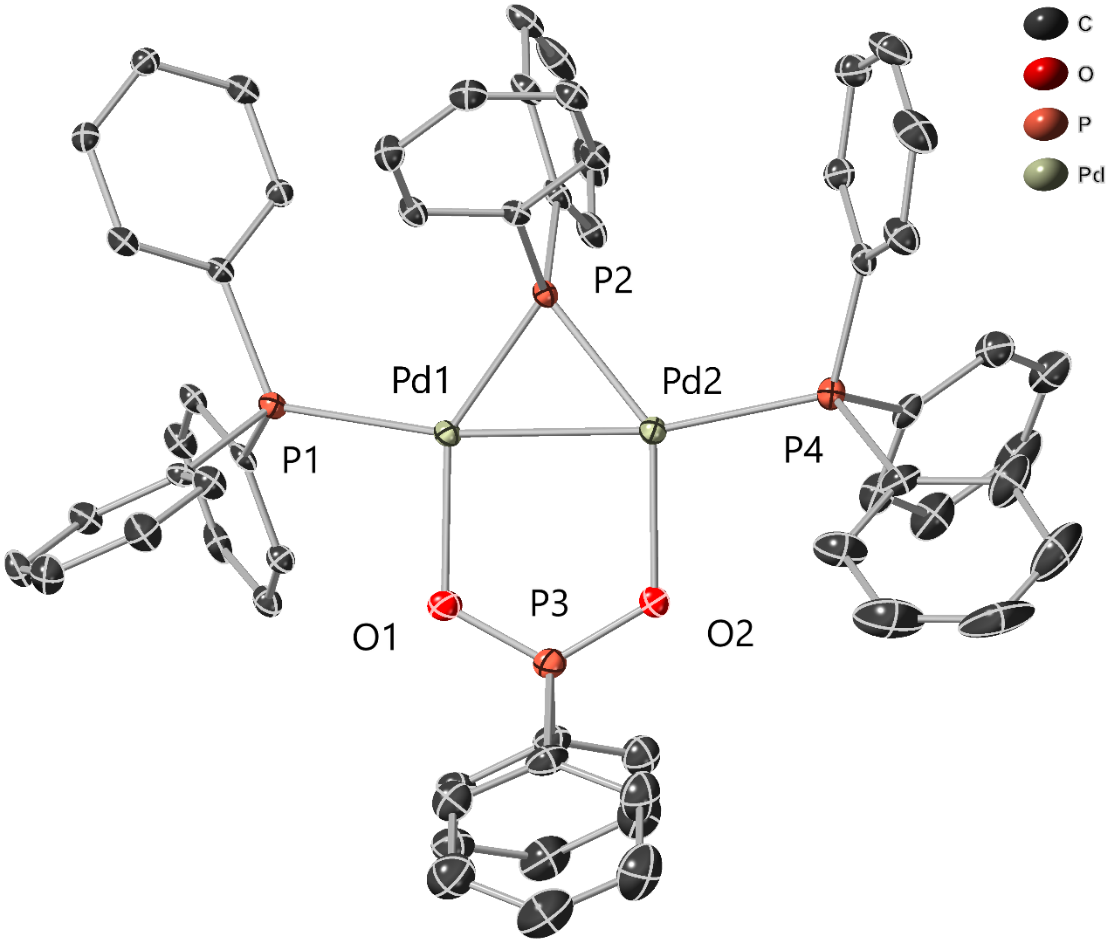

Figure 12 X-ray crystal structure of [Pd_2(μ-PPh_2){κ$_2$-P,O-μ-P(O)Ph_2}(κ-PPh_3)$_2$] (3)

Table S2 X-ray diffraction data for $[Pd_2(\mu\text{-}PPh_2)\{\kappa_2\text{-}P,O\text{-}\mu\text{-}P(O)Ph_2\}(\kappa\text{-}PPh_3)_2]$ (3)

**Empirical formula**	$C_{54}H_{51}O_3P_3Pd_2$
**Formula weight**	1053.66
**Temperature/K**	110.00(10)
**Crystal system**	monoclinic
**Space group**	$P2_1/c$
**a/Å**	11.8269(2)
**b/Å**	29.4867(5)
**c/Å**	14.1794(2)
**α/°**	90
**β/°**	108.7289(19)
**γ/°**	90
**Volume/Å^3**	4683.01(14)
**Z**	4
**ρ_{calc}g/cm^3**	1.494
**μ/mm^{-1}**	7.503
**F(000)**	2144.0
**Crystal size/mm^3**	0.258 × 0.093 × 0.018
**Radiation**	CuKα (λ = 1.54184)
**2Θ range for data collection/°**	7.234 to 134.156
**Index ranges**	-14 ≤ h ≤ 12, -19 ≤ k ≤ 35, -16 ≤ l ≤ 15
**Reflections collected**	17077
**Independent reflections**	8345 [R_{int} = 0.0266, R_{sigma} = 0.0369]
**Data/restraints/parameters**	8345/20/577
**Goodness-of-fit on F^2**	1.019
**Final R indexes [I>=2σ (I)]**	R_1 = 0.0271, wR_2 = 0.0620
**Final R indexes [all data]**	R_1 = 0.0345, wR_2 = 0.0658
**Largest diff. peak/hole / e Å^{-3}**	0.49/-0.52
**CCDC number**	1894929

Refinement Special Details

The THF of crystallization was disordered about the center of inversion with 0.5 molecules per asymmetric unit. C-C distances were restrained to be 1.50 angstroms and the C-O distances to 1.42 angstroms. The ADP of C61 and C64 were restrained to be approximately isotropic.

4.0. Computational studies: collated energies for complex 2

Density functional theory (DFT) methods were used to probe the structure of complex **2**. All calculations were performed at the DFT level using the B3LYP functional[8-10] in the Gaussian16(revision A.03) suite of programs.[11] The def2-SVP basis set was used for all atoms,[12,13] along with the SMD continuum model for tetrahydrofuran (ε= 7.4257) as an implicit solvent.[14] A superfine integration grid for all atoms was used throughout. The D3 version of Grimme's dispersion corrections with Becke-Johnson damping[15] were used throughout. The nature of stationary points was verified by frequency calculations, with minima having zero imaginary frequencies.

```
Zero-point correction=                           0.923255(Hartree/Particle)
Thermal correction to Energy=                    0.985919
Thermal correction to Enthalpy=                  0.986863
Thermal correction to Gibbs Free Energy=         0.812272
Sum of electronic and zero-point Energies=     -4010.389753
Sum of electronic and thermal Energies=        -4010.327089
Sum of electronic and thermal Enthalpies=      -4010.326145
Sum of electronic and thermal Free Energies=   -4010.500736
```

4.1. NBO and Wiberg indices for complex (2)

Table S3 Natural charges of central Pd and P motif

Atom	Number	Natural charge	Natural Population (Valence)	Natural Population (Total)
O	111	-1.03692	7.02823	9.03692
P	112	0.95885	3.98255	14.04115
P	113	0.65119	4.28775	14.34881
P	114	1.33250	3.57306	13.66750
P	115	0.98384	3.95715	14.01616
Pd	116	-0.29871	10.29913	46.29871
Pd	117	-0.04577	10.04472	46.04577

The O 111 atom has a formal -1 charge, and there is no negative charge allocated to the P 113, as would be expected for this complex.

Table S4 Calculated electron configuration of the atoms in the central motif

Atom	Number	Electron Configuration
O	111	[core] 2S(1.77) 2p(5.26) 3p(0.01)
P	112	[core] 3S(1.20) 3p(2.78) 4S(0.01) 3d(0.03) 4p(0.02)
P	113	[core] 3S(1.28) 3p(3.01) 4S(0.01) 3d(0.03) 4p(0.02)
P	114	[core] 3S(1.13) 3p(2.44) 4S(0.01) 3d(0.06) 4p(0.03)
P	115	[core] 3S(1.19) 3p(2.77) 4S(0.01) 3d(0.03) 4p(0.02)
Pd	116	[core] 5S(0.48) 4d(9.47) 5p(0.35) 5d(0.01)
Pd	117	[core] 5S(0.42) 4d(9.34) 5p(0.29) 5d(0.01)6p (0.01)

Table S5 Calculated Wiberg bond indices for atoms in the central motif

		111	112	113	114	115	116	117
		**O**	**P**	**P**	**P**	**P**	**Pd**	**Pd**
**111**	**O**	0	0.005	0.1018	**0.9477**	0.0186	0.0644	**0.2697**
**112**	**P**	0.005	0	0.059	0.0286	0.0303	**0.4581**	0.0613
**113**	**P**	0.1018	0.059	0	0.106	0.0721	**0.5573**	**0.6542**
**114**	**P**	0.9477	0.0286	0.106	0	0.0284	**0.4697**	0.1624
**115**	**P**	0.0186	0.0303	0.0721	0.0284	0	0.072	**0.4498**
**116**	**Pd**	0.0644	0.4581	0.5573	0.4697	0.072	0	**0.3167**
**117**	**Pd**	0.2697	0.0613	0.6542	0.1624	0.4498	0.3167	0

The relevant bonds for the central motif have been colored red. There is a significant bond calculated to be between P 113 and the two Pd atoms, but only a small interaction between O 111 and Pd 117.

Table S6 Calculated Wiberg total bonds by atoms

Atom	Number	Wiberg total bonds
O	111	1.6054
P	112	3.5574
P	113	3.6341
P	114	3.5826
P	115	3.5886
Pd	116	2.2837
Pd	117	2.2325

The Wiberg total bond of 1.6 for O 111 implies a partial bond between O 111 and Pd 117. All P atoms are calculated to have partial (0.5) bonds to the Pd atoms.

Table S7 Natural Atomic Orbital (NAO) bond orders (idealised Lewis structure)

		**111**	**112**	**113**	**114**	**115**	**116**	**117**
		**O**	**P**	**P**	**P**	**P**	**Pd**	**Pd**
**111**	**O**	0	0	-0.0047	0.8277	0.0057	0.0321	0.2308
**112**	**P**	0	0	0.0311	0.013	0	0.5526	0.0206
**113**	**P**	-0.005	0.0311	0	0.0015	0.0518	0.6142	0.6216
**114**	**P**	0.8277	0.013	0.0015	0	-0.0045	0.5684	0.2454
**115**	**P**	0.0057	0	0.0518	-0.005	0	0.0149	0.5337
**116**	**Pd**	0.0321	0.5526	0.6142	0.5684	0.0149	0	0.4737
**117**	**Pd**	0.2308	0.0206	0.6216	0.2454	0.5337	0.4737	0

Table S8 NAO bond order totals

Atom	Number	Bond order total
O	111	1.0640
P	112	3.1444
P	113	3.0233
P	114	3.2643
P	115	3.1312
Pd	116	2.6779
Pd	117	2.5728

Table S9 Natural Bond Orbitals information (bonding only)

Atom 1	Atom 2	Occupancy	Energy
O 111	P 114	1.98193	-0.82058
P 113	Pd 116	1.74116	-0.37201
P 113	Pd 117	1.77444	-0.24137
P 114	Pd 116	1.71374	-0.35895
P 115	Pd 117	1.88656	-0.40875

O 111 is calculated to have 3 lone pairs (occupancy 1.85500, 1.73775, 1.97655), indicating that there are only weak NBO bonding interactions between Pd 117 and O 111.

5. References

1. Scott, N. W. J.; Ford, M. J.; Schotes, C.; Parker, R. R.; Whitwood, A. C.; Fairlamb, I. J. S. The ubiquitous cross-coupling catalyst system '$Pd(OAc)_2$'/$2PPh_3$ forms a unique dinuclear Pd^{I} complex: an important entry point into catalytically competent cyclic Pd_3 clusters. *Chem. Sci.* **2019**, *10*, 7898-7906.
2. Coulson, D. R. Ready cleavage of triphenylphosphine. *Chem. Commun.* **1968**, 1530-1531.
3. Amatore, C.; Jutand, A.; M'Barki, M. A. Evidence of the Formation of Zerovalent Palladium from $Pd(OAc)_2$, and Triphenylphosphine. *Organometallics* **1992**, *11*, 3009-3013.
4. Berenblyum, A. S.; Aeeva, A. P.; Lakhman, L. I.; Moiseev, I. I. Mechanism of the formation of palladium complexes serving as catalysts in hydrogenation reactions. *J. Organomet. Chem.* **1982**, *234*, 237-248.
5. Dixon, K. R.; Rattray, A. D. Trinuclear palladium clusters: synthesis and phosphorus-31 nuclear magnetic resonance spectra of $[Pd_3Cl(PPh_2)_2(PPh_3)_3][BF_4]$ and related complexes. *Inorg. Chem.* **1978**, *17*, 1099-1103.
6. Fitton, P.; Rick, E. A. The addition of aryl halides to tetrakis(triphenylphosphine)palladium(0). *J. Organomet. Chem.* **1971**, *28*, 287-291.
7. Beeby, A.; Bettington, S.; Fairlamb, I. J. S.; Goeta, A. E.; Kapdi, A. R.; Niemelä, E. H.; Thompson, A. L. A new precatalyst for the Suzuki reaction—a pyridyl-bridged dinuclear palladium complex as a source of mono-ligated palladium(0). *New J. Chem.* **2004**, *28*, 600-605.
8. Lee, C.; Yang, W.; Parr, R. G. Development of the Colle-Salvetti correlation-energy formula into a functional of the electron density. *Phys. Rev. B.* **1988**, *37*, 785–789.
9. Becke, A. D., Density-functional thermochemistry. III. The role of exact exchange. *J. Chem. Phys.* **1993**, *98*, 5648–5652.
10. Stephens, P. J.; Devlin, F. J.; Chabalowski, C. F.; Frisch, M. J. Ab Initio Calculation of Vibrational Absorption and Circular Dichroism Spectra Using Density Functional Force Fields. *J. Phys. Chem.* **1994**, *98*, 11623–11627.
11. Gaussian 16, Revision A.03, Frisch, M. J.; Trucks, G. W.; Schlegel, H. B.; Scuseria, G. E.; Robb, M. A.; Cheeseman, J. R.; Scalmani, G.; Barone, V.; Petersson, G. A.; Nakatsuji, H.; Li, X.; Caricato, M.; Marenich, A. V.; Bloino, J.; Janesko, B. G.; Gomperts, R.; Mennucci, B.; Hratchian, H. P.; Ortiz, J. V.; Izmaylov, A. F.; Sonnenberg, J. L.; Williams-Young, D.; Ding, F.; Lipparini, F.; Egidi, F.; Goings, J.; Peng, B.; Petrone, A.; Henderson, T.; Ranasinghe, D.; Zakrzewski, V. G.; Gao, J.; Rega, N.; Zheng, G.; Liang, W.; Hada, M.; Ehara, M.; Toyota, K.; Fukuda, R.; Hasegawa, J.; Ishida, M.; Nakajima,

T.; Honda, Y.; Kitao, O.; Nakai, H.; Vreven, T.; Throssell, K.; Montgomery, J. A., Jr.; Peralta, J. E.; Ogliaro, F.; Bearpark, M. J.; Heyd, J. J.; Brothers, E. N.; Kudin, K. N.; Staroverov, V. N.; Keith, T. A.; Kobayashi, R.; Normand, J.; Raghavachari, K.; Rendell, A. P.; Burant, J. C.; Iyengar, S. S.; Tomasi, J.; Cossi, M.; Millam, J. M.; Klene, M.; Adamo, C.; Cammi, R.; Ochterski, J. W.; Martin, R. L.; Morokuma, K.; Farkas, O.; Foresman, J. B.; Fox, D. J. Gaussian, Inc., Wallingford CT, 2016.

12. Weigend, F. Accurate Coulomb-fitting basis sets for H to Rn. *Phys. Chem. Chem. Phys.* **2006**, *8*, 1057–1065.
13. Weigend, F.; Ahlrichs, R. Balanced basis sets of split valence, triple zeta valence and quadruple zeta valence quality for H to Rn: Design and assessment of accuracy. *Phys. Chem. Chem. Phys.* **2005**, *7*, 3297–3305.
14. Marenich, A. V.; Cramer, C. J.; Truhlar, D. G. Universal solvation model based on solute electron density and on a continuum model of the solvent defined by the bulk dielectric constant and atomic surface tensions. *J. Phys. Chem. B.* **2009**, *113*, 6378–6396.
15. Grimme, S.; Ehrlich, S.; Goerigk, L. Effect of the damping function in dispersion corrected density functional theory. *J. Comput. Chem.* **2011**, *32*, 1456–1465.
